# SexAnnoDB, a knowledgebase of sex-specific regulations from multi-omics data of human cancers

**DOI:** 10.1186/s13293-024-00638-8

**Published:** 2024-08-22

**Authors:** Mengyuan Yang, Yuzhou Feng, Jiajia Liu, Hong Wang, Sijia Wu, Weiling Zhao, Pora Kim, Xiaobo Zhou

**Affiliations:** 1 School of Life Sciences, Zhengzhou University, Zhengzhou, 450001 China; 2 West China Biomedical Big Data Center, West China Hospital, Sichuan University, Chengdu, 610041 China; 3 Shihezi University School of Medicine, Shihezi University, Shihezi , 832003 China; 4 Center for Computational Systems Medicine, McWilliams School of Biomedical Informatics, The University of Texas Health Science Center at Houston, Houston, 77030 USA; 5 School of Life Sciences and Technology, Xidian University, Xi’an, 710126 China

**Keywords:** Sex difference, Cancer, Multi-omics, Sex-biased regulatory network

## Abstract

**Background:**

Sexual differences across molecular levels profoundly impact cancer biology and outcomes. Patient gender significantly influences drug responses, with divergent reactions between men and women to the same drugs. Despite databases on sex differences in human tissues, understanding regulations of sex disparities in cancer is limited. These resources lack detailed mechanistic studies on sex-biased molecules.

**Methods:**

In this study, we conducted a comprehensive examination of molecular distinctions and regulatory networks across 27 cancer types, delving into sex-biased effects. Our analyses encompassed sex-biased competitive endogenous RNA networks, regulatory networks involving sex-biased RNA binding protein-exon skipping events, sex-biased transcription factor-gene regulatory networks, as well as sex-biased expression quantitative trait loci, sex-biased expression quantitative trait methylation, sex-biased splicing quantitative trait loci, and the identification of sex-biased cancer therapeutic drug target genes. All findings from these analyses are accessible on SexAnnoDB (https://ccsm.uth.edu/SexAnnoDB/).

**Results:**

From these analyses, we defined 126 cancer therapeutic target sex-associated genes. Among them, 9 genes showed sex-biased at both the mRNA and protein levels. Specifically, *S100A9* was the target of five drugs, of which calcium has been approved by the FDA for the treatment of colon and rectal cancers. Transcription factor (TF)-gene regulatory network analysis suggested that four TFs in the SARC male group targeted *S100A9* and upregulated the expression of *S100A9* in these patients. Promoter region methylation status was only associated with *S100A9* expression in KIRP female patients. Hypermethylation inhibited *S100A9* expression and was responsible for the downregulation of *S100A9* in these female patients.

**Conclusions:**

Comprehensive network and association analyses indicated that the sex differences at the transcriptome level were partially the result of corresponding sex-biased epigenetic and genetic molecules. Overall, SexAnnoDB offers a discipline-specific search platform that could potentially assist basic experimental researchers or physicians in developing personalized treatment plans.

**Supplementary Information:**

The online version contains supplementary material available at 10.1186/s13293-024-00638-8.

## Plain language summary

Sexual variations at the molecular level have a profound impact on cancer biology and outcomes, influencing drug responses that diverge between men and women exposed to the same drugs. Despite existing databases on sex differences in human tissues, our understanding of the regulations governing sex disparities in cancer is limited, lacking detailed mechanistic studies on sex-biased molecules. This study addresses this gap by conducting a comprehensive examination of molecular distinctions and regulatory networks across 27 cancer types, specifically focusing on sex-biased effects. The analyses led to the identification of 126 cancer therapeutic target sex-associated genes and shed light on the intricate relationship between sexual differences and cancer. Furthermore, the findings from these analyses are made accessible through SexAnnoDB, providing a specialized search platform. This platform has the potential to assist basic experimental researchers or physicians in developing personalized treatment plans based on a deeper understanding of sex-specific factors in cancer.

**Supplementary Information:**

The online version contains supplementary material available at 10.1186/s13293-024-00638-8.

## Highlights

- SexAnnoDB identified 4,328 genes exhibiting sex-biased signatures, with 126 of these genes being implicated as cancer therapeutic targets.
- SexAnnoDB provided a comprehensive examination of regulatory networks across 27 cancer types, delving into sex-biased effects.
- SexAnnoDB is the first one to provide sex-biased alternative splicing regulation and offer potential sex-related alternative splicing markers.
- SexAnnoDB offers a discipline-specific search platform that could potentially assist basic experimental researchers or physicians in developing personalized treatment plans.

**Supplementary Information:**

The online version contains supplementary material available at 10.1186/s13293-024-00638-8.

## Background

Accumulating evidence indicates that the difference in genetic and molecular characteristics between males and females affects cancer incidence, prognosis, and treatment responses [1, 2]. For example, females carrying the MDM2 SNP309G variant, who evade cancer, uniquely experience extended longevity. This aligns with elevated MDM2 levels, which weaken the effectiveness of the p53 stress response but prolong the period of stem cell repopulation among survivors. This correlation was observed in female centenarians carrying one or two copies of this allele, while there was no discernible association among males [3, 4]. An essential observation is the association between sex disparity and the methylation status of genes encoding components of the p53 pathway. This relationship is particularly evident in gastric cancer cases at stages II and III, underscoring the peril posed by heightened methylation levels of p53 pathway genes. Specifically, within a wild-type TP53 context, males exhibit a heightened risk, correlated with the methylation-induced disruption of three genes within the p53 pathway. In contrast, females demonstrate lower rates of methylation, consequently experiencing a reduced risk of gastric cancer [5]. Several studies have successfully identified differential sex-biased molecular patterns using muti-omics data [6–9]. Currently, there are several studies on sex differences in human tissues including GendermedDB, SDC, SAGD, and Janusmed Sex and Gender [8, 10–13]. GendermedDB provided a search engine for sex and gender-specific publications. SAGD provided the sex-associated genes from transcriptomes of 21 species. SDC provides survival and phenotype, molecular differences, signatures, enrichment pathways, and therapy response. Janusmed Sex and Gender provides information about more than 400 drug substances within several therapeutic areas. While much research has focused on understanding the influence of gene expression levels and DNA mutation on sex-specific adaptation, other regulatory mechanisms have received less attention. Abnormal regulation of alternative splicing and RNA editing, often dysregulated in cancer, has been highlighted in numerous studies. In our research, we not only explore sex-biased gene expression regulation but also analyze sex-biased alternative splicing regulation and RNA editing events. Through comprehensive analyses of sex-biased regulation, we have developed SexAnnoDB, a user-friendly website tailored to researchers investigating cancer and sex differences. SexAnnoDB serves as a valuable resource and reference for extensive annotations of sex-difference-related regulations of various molecules in cancer. This study aims to advance the development of gender-related personalized precision medicine for cancer patients.

To comprehend the landscape of regulatory sex differences between males and females, we conducted a comprehensive analysis of molecules at various levels across different cancer types. Our study focused on cancer types with a minimum of five patients in each gender group, as per The Cancer Genome Atlas (TCGA). The molecules examined spanned multiple levels, encompassing the genome (single nucleotide variants, SNVs, and single nucleotide polymorphisms, SNPs), the epigenome (DNA methylation), the transcriptome (mRNA, lncRNA, miRNA, exon skipping events, and RNA editing events), and the proteome (protein). For each molecular signature, we employed multiple systematic and bioinformatic analyses. These included investigations into sex-specific differential expression, sex-biased expression, sex-related transcription factor (TF)-gene expression regulations (TF-gene regulations), sex-related RNA binding protein (RBP)-exon skipping (ES) regulations (RBP-ES regulations), sex-biased competing endogenous RNA regulations (ceRNAs), and various types of sex-biased quantitative trait loci studies.

**Fig. 1:**
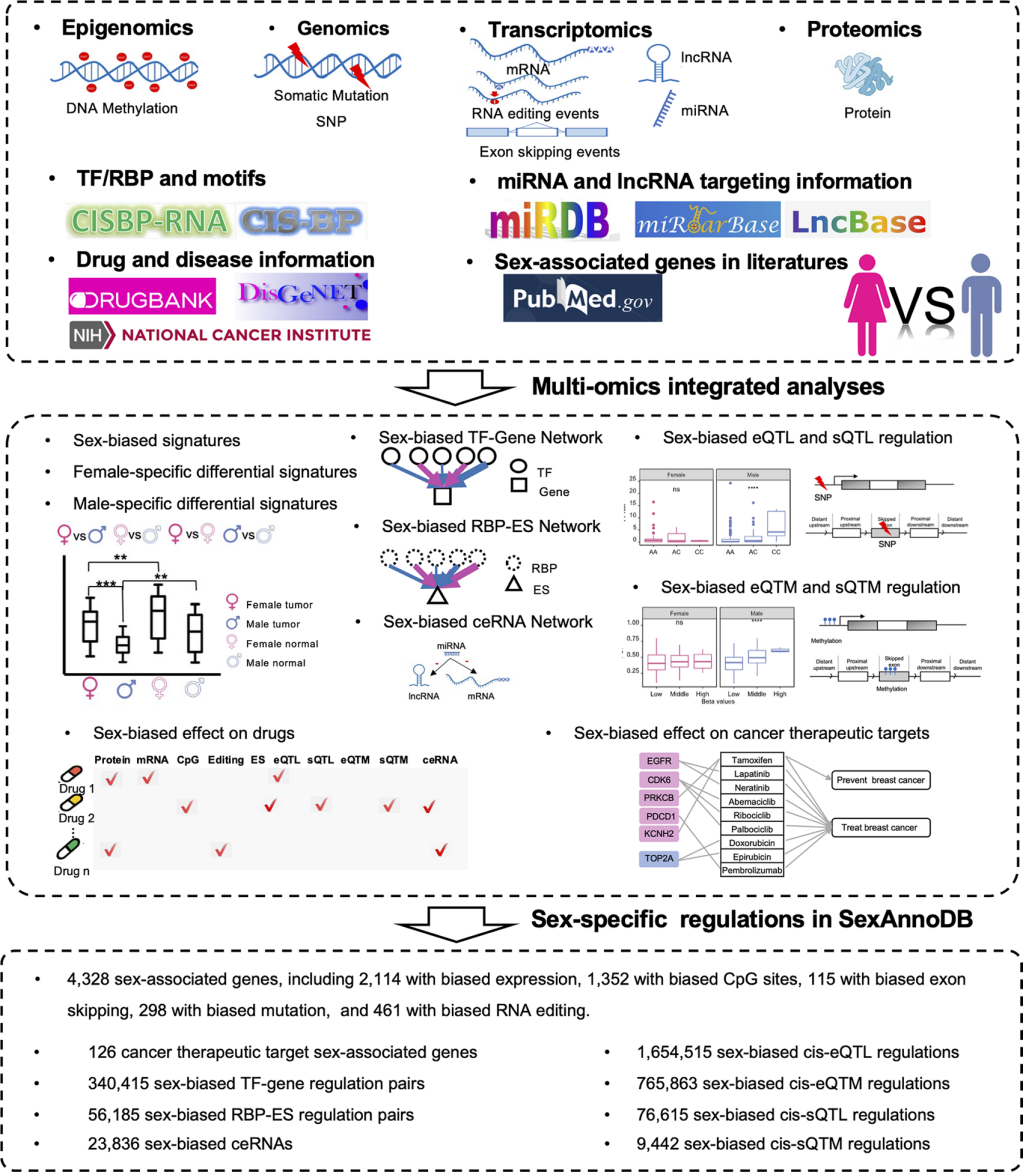
Overview of SexAnnoDB. The molecules used to explore the sex difference in cancer include the genome level (single nucleotide variant, single-nucleotide polymorphisms), epigenome level (DNA methylation), transcriptome level (mRNA, lncRNA, miRNA, exon skipping events, and RNA editing events), and proteome level (protein). The analyses performed to explore the sex-biased regulations in cancer include differential analysis for each condition group, sex-biased network analysis, sex-biased quantitative trait loci studies, functional enrichment analysis, and drug-disease information. For detailed information on sex-related regulations, SexAnnoDB offers accessible and downloadable results. Visit https://ccsm.uth.edu/SexAnnoDB/ for more details

From our analyses, we identified 4,328 genes exhibiting sex-biased signatures, with 126 of these genes being implicated as cancer therapeutic targets. In the realm of sex-related regulation, we scrutinized 340,415 sex-biased TF-gene regulation pairs, 56,185 sex-biased RBP-ES regulation pairs, and 23,836 sex-biased ceRNAs. Additionally, we examined 1,654,515 sex-biased expression quantitative trait loci (eQTL) pairs, 76,615 sex-biased splicing quantitative trait loci (sQTL) pairs, 765,863 sex-biased expression quantitative trait methylation (eQTM) pairs, and 9,442 sex-biased splicing quantitative trait methylation (sQTM) pairs to elucidate variant and methylation effects at the transcriptome level (Fig. 1). Subsequently, for the identified 4,328 genes exhibiting sex-biased signatures, we conducted manual curation of PubMed articles to collate sex-related reports from previous studies. All such information is accessible and downloadable on the SexAnnoDB website (https://ccsm.uth.edu/SexAnnoDB).

## Materials and methods

### Data preparation

To explore the sex differences in cancer, we first downloaded the multi-omics data and clinical data from the cancer genome atlas (TCGA, https://portal.gdc.cancer.gov/) of 27 cancer types [14]. For further analysis, we categorized the patients into four groups: male tumor samples, female tumor samples, male normal samples, and female normal samples based on gender information. Only the group has at least five patients saved for further analysis (Supplementary Fig. S1A, Supplementary Table 1). Then, we collected alternative splicing events information on TCGA from the study by Kahles et al. (https://gdc.cancer.gov/about-data/publications/PanCanAtlas-Splicing-2018) [15]. Finally, RNA editing events information from CAeditome [16] was downloaded to study the sex difference of RNA editing between the male and female tumor groups.

### Analysis somatic mutation data

We obtained the single nucleotide variants data (MAF files) and somatic copy number variations (CNVs) from TCGA. First, filter out the samples with > 1,000 mutations in their exomes. Only mutations with mutation frequency bigger than 5% were using mafCompare() to identify the sex-biased mutation with p-value < 0.05 [17]. Then, we conducted functional enrichment analysis using Enrichr [18] to infer potentially involved biological pathways of sex-biased mutation genes.

### Analysis of DNA methylation

Wilcoxon test and the following Benjamini-Hochberg false discovery rate for multiple testing were applied for CpG sites. Differentially methylated CpGs were reported if the mean methylation difference was > 0.1 with a p.adjuest of 5%. We also annotated the CpG sites into a promoter, enhancer, coding sequence region (CDS), untranslated regions (UTR), and gene body region based on the position of CpG sites.

### Analysis of gene/lncRNA/miRNA expression

For the analysis of gene/lncRNA/miRNA expression, we filter out the genes that have missing values in more than 20% of patients and require an average expression bigger than 1 of each cancer type. For each compare group, we ran DESeq2 [19] and performed differentially expressed lncRNAs/mRNAs/miRNAs analyses of individual cancer types (|log2FC|>1 and p.adjusted < 0.05).

### Analysis of RNA A-to-I editing events

Differential RNA editing events were identified based on significantly different editing frequencies (*P* < 0.05) determined using the Wilcoxon test, followed by multiple testing correction using the Benjamini-Hochberg false discovery rate. Subsequently, we utilized the annotation file from CAeditome to assess the deleterious effects of RNA editing events on protein functions and RNA ending effects to miRNAs.

### Analysis of exon skipping events

We first excluded exon skipping events with missing values in over 20% of patients and required an average gene expression with exon skipping events exceeding 1 for each cancer type. Then, we applied the Wilcoxon test to analyze exon skipping events, followed by Benjamini-Hochberg false discovery rate correction for multiple testing. Differential ES events were identified if the mean differential percent spliced in (PSI) value was greater than 0.1, with a p.adjust of 5%. To assess the effects of exon skipping on open reading frames (ORF) and protein functions, we utilized the annotation file from ExonskipDB [20].

### Analysis of protein expression

The Wilcoxon test and the following Benjamini-Hochberg false discovery rate for multiple testing were applied for CpG sites, ES events, RNA editing events, and proteins. For the differential protein, we require the p.adjuest < 0.05.

### Define sex-specific differential signatures and sex-biased signatures in cancer

Sex-specific differential signatures in cancer indicate molecular changes between tumor and normal tissues based on patient gender. Male-specific molecules vary in male tumor vs. normal tissues, while female-specific molecules vary in female tissues. Sex-biased signatures differ only between male and female tumors (Supplementary Fig. S1B). Then, we conducted functional enrichment analysis using Enrichr [18] to identify potential biological pathways associated with these gender-specific molecules.

### Sex-biased expression quantitative trait loci (eQTL) and sex-biased exon skipping-specific splicing quantitative trait loci (sQTL)

A genotype was removed for each SNP site if its read depth (DP) was less than 10 or genotype quality (GQ) was less than 20. Additionally, each SNP should be bi-allelic. Then we filter out variants that did not meet the Hardy–Weinberg equilibrium (HWE-*P* > 10 − 6); and low minor allele frequency (MAF < 0.5). Furthermore, the quantitative trait loci studies require no less than 50 patients. To identify the sex-biased cis-eQTLs and sex-biased cis-sQTLs, we first filtered out the low expressed gene (FPKM < 1). Then, the R package MatrixeQTL 2.3 [21] was used to perform the eQTL/sQTL analysis and required FDR < 0.05. To control potential confounding factors such as age of death, age_at_index, race, and age_at_diagnosis, we included these variables in the analysis as covariates. Cis-eQTLs and cis-sQTLs were defined if the SNP were within 100 kb from the gene transcriptional start site (TSS) or skipped exon region (Supplementary Figs. S2,S3). Finally, the sex-biased cis-eQTLs/cis-sQTLs were selected if only significant in one sex group or showed opposite regulations between male and female patients.

### Sex-biased exon skipping-specific splicing quantitative trait methylation(sQTM) and sex-biased expression quantitative trait methylation(eQTM)

To identify the sex-biased cis-sQTMs and sex-biased cis-eQTMs, we first categorized the patients into three groups based on the beta value(β) of each CpG site, including low methylation (0 ~ 0.2), middle methylation (0.2 ~ 0.6), and high methylation (0.6 ~ 1.0) groups. Then R package MatrixeQTL was used to identify the sQTM pairs and eQTM pairs and required FDR < 0.05. To control potential confounding factors such as age of death, age_at_index, race, and age_at_diagnosis, we included these variables in the analysis as covariates. Here, we defined cis-sQTM/cis-eQTM as CpG sites located in [-100 kb, 100 kb] region of TSS and ES events. Then, we categorized the CpG sites into distant upstream, proximal upstream, skipped exon region, proximal downstream, and distant downstream groups based on the position of CpG sites. Finally, the sex-biased cis-eQTMs/cis-sQTMs were selected if the cis- eQTMs/cis-sQTMs only significant in one sex group or showed opposite regulations between male and female patients (Supplementary Fig. S3).

### Analysis of competitive endogenous RNAs(ceRNAs) network

To explore the sex-biased ceRNA regulations, we predicted lncRNA–miRNA interaction pairs based on LncBase v3.0 [22] predicted databases. Subsequently, miRTarBase Release 9.0 [23], miRDB 6.0 [24], and TargetScan huam 8.0 [25] databases were used to identify miRNA–mRNA interaction pairs. Target mRNAs recognized by at least two databases were selected as candidate genes. Finally, we integrated the interaction between sex-biased miRNAs and sex-biased lncRNAs or sex-biased mRNAs to construct a sex-biased ceRNA regulatory network.

### Analysis of transcription factor-gene regulatory network

We used PANDA [26] in R package to infer gene regulatory network models on each condition group of each cancer type. To compare those network models between different condition groups, we used the function panda.diff.edges() in and 0.98 as the threshold for the differential edges [27]. Specifically, we first downloaded position weight matrices (PWMs) for Homo sapiens motifs of TFs from CIS-BP 2.0 [28] and mapped PWMs to gene promoter regions using FIMO 5.5.3 (p-value < 1e-4) [29]. Then, PANDA was used to estimate and optimize the network’s structure based on integrating TF binding motifs, gene expression profiles, and protein-protein interactions. The protein-protein interaction (PPI) data were generated as described by Sonawane et al [30]. To generate the PPI data, the interactions between all TFs in the regulatory prior were obtained and weighted based on interaction scores from StringDB v11.5 [31]. In detail, the PPI data was estimated between all TFs using interaction scores from StringDb v11.5, which were scaled to be within a range of [0,1], where self-interactions were set equal to one. Finally, sex-biased TF-gene edges were selected based on the differential edges analysis, one female-biased TF-gene edge was identified if the threshold of the female tumor network was bigger than 0.98 and the threshold of the male tumor was no bigger than 0.98.

### Analysis of RNA binding protein-exon skip events regulatory network

We first mapped the Homo sapiens PWM motifs of RBPs from CisBP-RNA 0.6 [32] to skipped exon regions of exon skip events (Supplementary Fig. S2) using FIMO (p-value < 1e-4) to get the RBP-ES interaction. Then, the RBP-ES network for each condition group was constructed using three data types, including RBP binding motifs, PSI values of exon skip events and protein-protein interactions. Finally, we used the function panda.diff.edges() and 0.98 as a threshold for the differential edges to compare those network models between different condition groups.

### Identifying of sex-biased cancer therapeutic drug target gene

Firstly, drug-target interactions (DTIs) were extracted from DrugBank 5.0 [33], and duplicated DTI pairs were excluded. Then, we collected 205 cancer therapeutic target genes that were targeted by 206 drugs for 40 cancer types from the National Cancer Institute (https://www.cancer.gov/about-cancer/treatment/drugs/cancer-type). All drugs were grouped using Anatomical Therapeutic Chemical (ATC) classification system codes. Last, disease-genetic information was extracted from DisGeNET v7.0 [34]. Finally, 131 drug target genes were associated with at least one type of sex-biased signature, and 126 were cancer therapeutic target genes.

### Manual curation of PubMed articles

For 4,328 genes with sex-biased signatures, PubMed’s literature was performed on May 2024 using the search expression applied to each gene [35]. Taking S100A9 as an example, it is ‘((S100A9 [Title/Abstract]) AND sex difference [Title/Abstract]))’ and ‘((S100A9 [Title/Abstract]) AND sex difference [Title/Abstract]) AND (cancer [Title/Abstract]))’.

## Results

### Sex-biased expressed molecular affect cancer progression

**Fig. 2:**
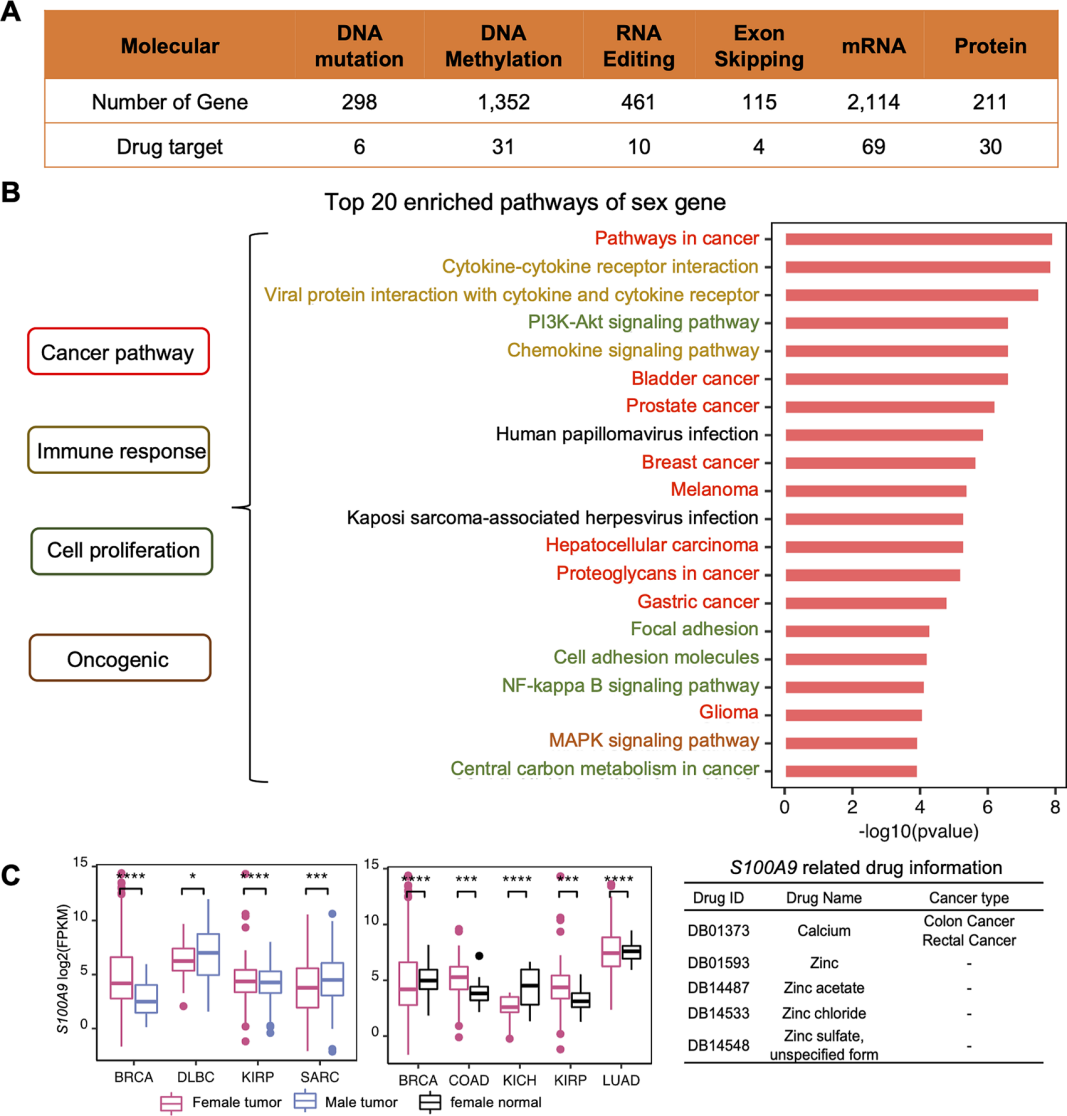
Sex-biased molecular signatures in human cancers. **(A)**The number of sex-biased signatures and number of sex-biased genes which are drug target. **(B)**Top 20 enriched KEGG pathways of genes which have at least one sex-biased signature. **(C)**From left to right are the expression of S*100A9* in four cancers, and pink represents the female tumor patients, blue represents the male tumor patients; the expression of *S100A9* five cancers, and pink represents the female tumor patients, black represents the female normal tissues. the drug and disease information of *S100A9*. Calcium has been approved by the FDA for the treatment of colon and rectal cancers(*p.adjusted < 0.05, **p.adjusted < 0.01, ***p.adjusted < 0.001, ****p.adjusted < 0.001)

To understand the landscape of sex differences between males and females, we performed multiple systematic and bioinformatic analyses, such as sex-biased expression and sex-specific differential expression. From our analyses, we defined 4,328 genes with sex-biased signatures, including 298 genes with sex-biased mutations, 1,352 genes with highly significant sex-biased CpG sites (|difference beta value| >0.3), 2,114 sex-biased mRNAs, and 115 genes with sex-biased exon skipping which affect the open reading frame, and 461 genes with sex-biased RNA editing affect coding regions (Fig. 2A, Supplementary Table 2). We performed functional enrichment analysis on 4,328 genes and identified several themes among the sex-affected pathways. The first group related to cancer and oncogenic signaling pathways, including multiple cancer types. The second group related to cancer immune response includes the chemokine signaling pathway, cytokine-cytokine receptor interaction, viral protein interaction with cytokine, and cytokine receptors. The third group was the cell proliferation-related pathway including growth-factor signaling, cell proliferation pathways such as focal adhesion, cell adhesion molecules, NF-kappa B signaling pathway, PI3K-Akt signaling pathway, and central carbon metabolism in cancer (Fig. 2B).

In this work, we identified significant differences in gene expression between male and female tumor tissues across multiple cancer types. Specifically, 2114 genes exhibited differential expression between male and female tumors, with 1593 genes showing tumor-specific patterns and 521 genes displaying consistent differential expression across several cancer types. Furthermore, we observed 6,979 genes with distinct expression profiles between female tumors and normal female tissue across all cancers studied. Among these, the number of genes showing female-specific differential expression varied widely across cancer types, ranging from 203 to 2,924. Similarly, between male tumors and male normal tissues, 298 to 4204 genes showed differential expression across the 13 cancer types analyzed (Supplementary Fig. S4). Take *S100A9* as an example, *S100A9* was the target of five drugs, of which the FDA has approved calcium for the treatment of colon and rectal cancers [36]. *S100A9* has been reported to regulate the behavior of cancer cells by inducing pre-metastatic cascades associated with cancer spread [37]. A previous study showed that *S100a9* protected male lupus-prone *NZBWF1* mice from disease development [38]. Our study found that *S100A9* was differentially expressed between male and female patients in four cancer types (BRCA, DLBC, KIRP, SARC). Furthermore, through differential analysis between male/female tumors and their matched normal counterparts, we found that the expression difference of *S100A9* between tumors and normal counterparts was also significantly different in the male and female groups (Fig. 2C). These results provide an overview of molecular differences between male and female cancer patients and imply sexual differentiation affects cancer incidence and progression.

### Sex-biased somatic mutation data and single nucleotide polymorphisms showed different regulatory patterns between females and males

**Fig. 3:**
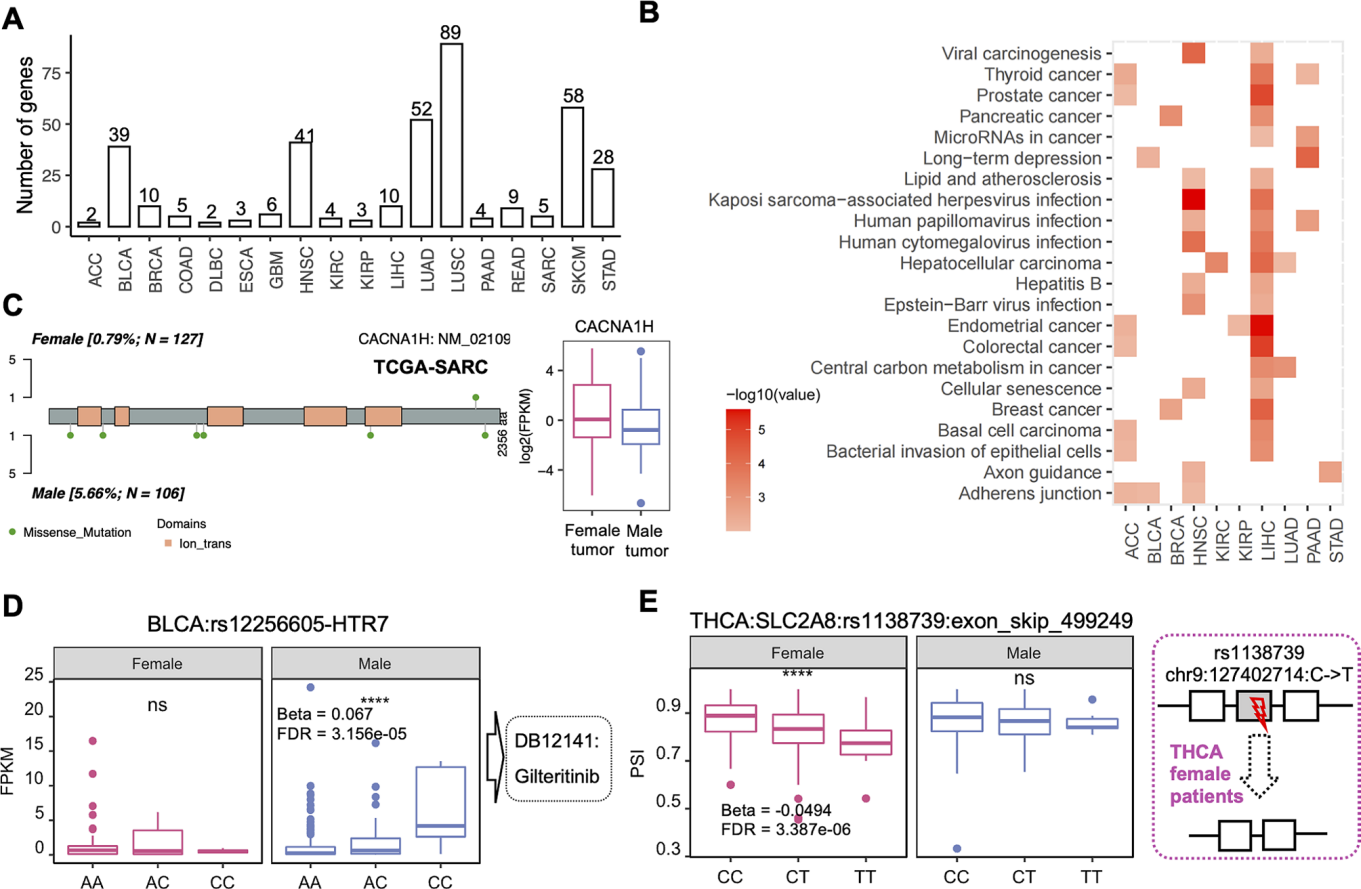
Sex-biased mutations**(A)** The number of gene with sex-biased mutation.**(B)** The functional enrichmentment result of gene with sex-biased mutation for each cancer type.**(C)** The sex-biased cis-eQTL of rs12256605 and HTR7 in BLCA. **(D)** The sex-biased cis-sQTL of rs12256605 and exon_skip_499249 in THCA.

Evaluating genetic variants based on sex can reveal important sex disparities. Sex-biased effects of variants related to cell cycle and apoptosis have been described for many cancer types. From our analysis, a total of 298 genes in 18 cancers show the differential frequency between male and female tumor patients. Functional enrichment analysis found only 10 cancers sex-biased genes enriched in cancer related pathways. For example, 10 mutation gene showed sex-biased frequency in LIHC. Among them, *CTNNB1*,* OBSCN*,* TP53*,* ALB* and *HERC2* show higher mutation frequency in male patients. *BAP1*,* PCLO*,* TENM1*,* PIK3CA*,* PIKCA*, and *PKHD1L1* show higher mutation frequency in female patients. These genes enriched in hepatocellular carcinoma, central carbon metabolism in cancer, cellular senescence and lipid and atherosclerosis (Fig. 3A-B, Supplementary Fig. S1A). By integrating with six-biased gene expression, 3 genes with sex-biased mutation frequency also showed differential expression between male and female patients. Specifically, a previous study report *CACNA1D* overexpression and activate voltage-gated calcium channels in prostate cancer during androgen deprivation [39]. *CACNA1D* has been identified as a potential therapeutic target. It is upregulated in pancreatic cancer cells and associated with tumor growth and invasion. In this work, *CACNA1D* was found to show high mutation frequency in male SARC patients with a down-regulation transcript level. Recognizing and addressing sex differences patterns is essential for delivering personalized and effective patient care (Fig. 3C, Supplementary Fig. S5B).

Furthermore, single nucleotide polymorphism is the simplest form of DNA variation among individuals [40]. They are responsible for the diversity of individuals, drug response, and complex and common diseases [41]. SNPs can affect alternative splicing by modifying the sequence-specific binding affinity of the splicing factors to the pre-mRNAs [42], resulting in splicing quantitative trait locus. And may influence promoter activity [40], resulting in the expression of quantitative trait locus. To explore the sex-biased effects of SNPs on gene expression and alternative splicing, we performed eQTL and sQTL analyses for the male and female patients, separately. 1,654,515 sex-biased cis-eQTLs pairs and 76,615 sex-biased cis-sQTL pairs were identified. Of 1,654,515 sex-biased cis-eQTLs, 214,335 were found to occur in 2,294 drug target genes including 254 cancer therapeutic targets (Supplementary Fig. S5C). Based on the position of the SNPs, we identified 578, 408, 163, 731, and 430 SNPs located in the promoter, enhancer, coding region (CDS), exon, and UTR of the gene, and 11,055 SNPs in the gene body region. For example, *HTR7* promoted laryngeal cancer growth through the activation of the PI3K/AKT pathway [43]. Rs12256605(chr10:9086171, G to C) located in the promoter region of *HTR7* showed a significant correlation with the expression of *HTR7* only in male BLCA (Fig. 3D). *HTR7* is the drug target of gilteritinib, the sex-based cis-eQTL regulation of *HTR7* may lead to different treatment effects of gilteritinib and will help to design personalized medicine.

Sex-biased sQTL analysis identified 76,615 sex-biased cis-sQTL pairs including 72,349 SNPs and 2,872 ES events in 2,256 genes. Furthermore, we overlapped the genomic coordinates of exon skipping events and SNPs and identified 17 SNPs located in the skipping regions of 14 ES events showing the sex-biased cis-sQTL regulations. For example, *SLC2A8* belongs to the solute carrier 2 A family [44]. The skipping of exon5 (exon_skip_499249, chr9:127402556–127402753) in *SLC2A8* was affected by rs1138739 in THCA female group. The mutation of rs1138739 in the skipped exon region may lead to exon skipping in female patients, resulting in frameshift ORF that may affect the cancer progression (Fig. 3E).

### DNA methylation-related sex-biased regulations

**Fig. 4:**
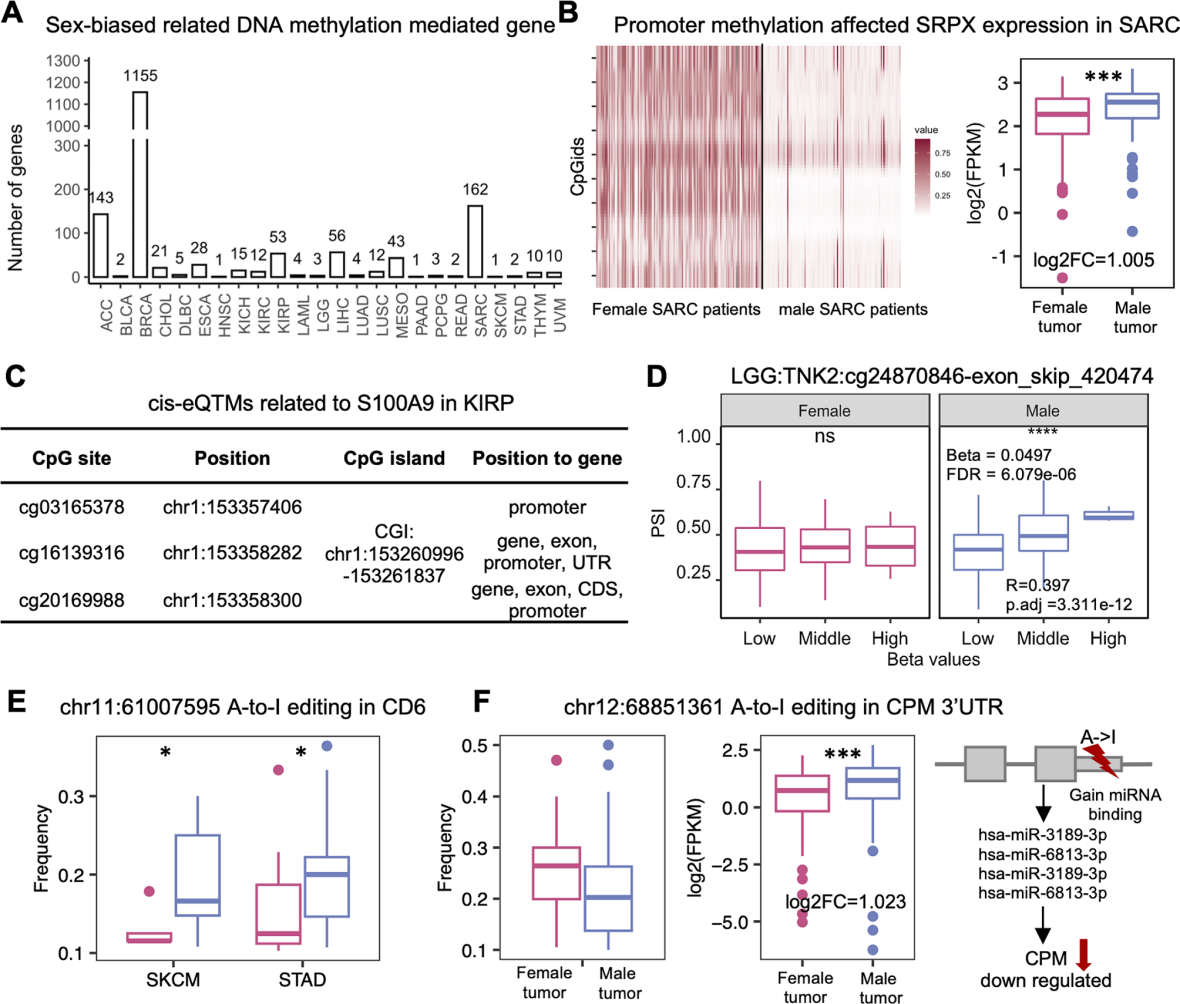
DNA methylation-related sex-biased regulations and Sex-biased RNA editing. **(A)**The number of sex-biased related DNA methylation mediated genes. **(B)** Promoter methylation affected SRPX expression in SARC. Left is the heatmap of 10 CpG sites in SRPX promoter regions. **(C)** CpG sites associated with S100A9 in KIRP. **(D)** The PSI values of exon_skip_499249 in low methylation (0 ~ 0.2), middle methylation (0.2 ~ 0.6), and high methylation (0.6 ~ 1) groups. The patients were categorized into three groups based on the beta value of cg24870846. **(E)** RNA editing frequence of chr11:61007595 in CD6. **(F)** chr12:68851361 A-to-I editing in CPM 3’UTR. From left to right are the RNA editing frequency of chr12:68851361; the expression of CPM; and the mechanism of miRNA binding increase in CPM 3’UTR region through RNA editing variant(*FDR < 0.05, ** FDR < 0.01, ***FDR < 0.001, ****FDR < 0.0001).

Methylation appears to influence gene expression and splicing patterns by affecting the interactions of DNA with both chromatin proteins and specific transcription factors [45, 46]. Combining the mRNA and DNA methylation analyses, we identified 1,672 sex-biased CpG sites in the gene promoter region of 582 sex-biased genes (Fig. 4A, Supplementary Table 3). For example, *SRPX* is a tumor-suppressor gene that was reported to be downregulated in a variety of human tumor cells and tissues. A previous study reported that *Srpx*-knockout mice generated various tumors, including lymphomas, lung cancer, and hepatomas [47, 48]. We found *SRPX* showed differential expression between male and female tumor patients. Our data suggested that total 10 CpG sites located in the *SRPX* promoter region showed higher methylation in female SARC patients inhibiting the expression of *SRPX* in female SARC patients (Fig. 4B, **Supplementary Fig. 6A)**. This result is consistent with the established role of DNA methylation in gene regulation that hypermethylation leads to gene silencing [49].

To further analyze the effect of sex-biased methylation site, we integrated multi-omics and performed eQTM and sQTM analyses for the male and female patients. Our analyses identified 764,720 sex-biased cis-eQTM and 9,442 sex-biased cis-sQTM. 1,305 sex-biased cis-eQTMs were found in 338 drug target genes, including 38 cancer therapeutic target genes (**Supplementary Fig. 5C**). Detailly, three CpG sites in CpG island (chr1:153260996–153261837) were associated with *S100A9* expression in the KIRP female group. The high methylation in the promoter region inhibits the expression of *S100A9*, which may influence *S100A9*-targeted drug resistance and cancer progression in female patients (Fig. 4C, Supplementary Fig. S6B).

Methylation at the DNA level can modulate the elongation rate of RNA polymerase II or the format of a protein bridge of splicing factor to affect the exon skipping [50]. This work identified 9,442 sex-biased cis-sQTMs, including 8,738 CpG sites and 2,008 ES events. Among them, 27 exon-skipping events occurred in the cancer therapeutic target genes. For example, *TNK2* was the target of entrectinib [33], which has been approved to treat non-small cell lung cancer and solid tumors in the body [51, 52]. Our results showed the inclusion of exon 14 in *TNK2* (exon_skip_420474, chr3:195867168–195867258) was positively correlated with cg24870846 in male LGG patients (Fig. 4D). Low methylation of cg24870846 could cause the in-frame ORF and result in the loss of the ‘UBA domain’, and ‘Activated CDC42 kinase 1 chain’ of *TNK2*. Overall, SexAnnoDB provides epigenetic factors that potentially influence gene expression and splicing. These factors may affect the downstream regulations of cancer progression. All filtered sex-biased cis-eQTMs and sex-biased cis-sQTMs were shown in SexAnnoDB.

### Sex-biased RNA editing affected the protein function and gene expression

RNA editing events in coding regions can alter amino acid sequences and have a chance to affect protein functions. To study this, we first identified 19,503 RNA editing site show difference frequency between male and female tumor patients. 14 of them in coding region might cause the non-synonymous SNV of 12 genes in 9 cancer types. Specifically, CD6 is a cell surface glycoprotein on human lymphocytes, including T cells and natural killer (NK) cells. recent studies strongly suggest that anti-CD6 should also be evaluated as a new cancer immunotherapy [53–55]. In this work, we found an RNA editing events in the position of chr11:61007595 show the differential frequency between male and female patients in two cancer types (SKCM, STAD). The RNA editing leads to the S/G changes of SRCRdomain in the CD6 protein (Fig. 4E, Supplementary Fig. S6C).

Combining the mRNA and RNA editing analyses, we identified 22 sex-biased RNA editing sites of 14 sex-biased genes(Supplementary Table3). Among them, 6 RNA editing sites were in 5 gene 3’UTR region. From the analysis of A-to-I RNA editing effects on miRNA regulation in CAeditome, 3 RNA editing will cause mRNA to gain miRNA binding sites, and 1 RNA editing will cause mRNA to lose miRNA binding sites (Supplementary Table S1). CPM can significantly inhibit cell viability, ROS production, intracellular pH, migration in hypoxic lung cancer cells, and angiogenesis of HUVECs under hypoxia through the inhibition of APEX1/HIF-1α interaction [56]. From our analysis, one RNA editing site in the CPM 3’UTR region shows higher editing frequency in SARC female patients. The editing alternation would cause the gain of four miRNAs binding and lead to downregulating its expression (Fig. 4F). Overall, the sex-biased RNA editing event has the potential to lead to protein loss of function or affect gene expression, which shows it’s potential to be a therapy target for precision treatment.

### RBP-ES regulatory networks identified the functional ES events related to cancer progression

RBPs are indeed primary regulators of splicing events. They interact with pre-mRNA molecules to facilitate or inhibit splicing, thereby influencing the production of mature mRNA transcripts [57]. PANDA is a method for estimating gene regulatory networks that were not specifically developed for RBP-ES networks, here we performed PANDA in 8 RBPs knockdown/out RNA-seq data to evaluate the performance of PANDA. We utilized RNA-seq and eCLIP data to identify the RBP-ES regulation pairs in RBP knocked. PANDA demonstrated moderate performance, achieving an AUC of 0.68 in identifying RBP-ES regulations (Supplementary Fig. 7, Supplementary method). Thus, in this work, we constructed RBP-ES networks using PANDA based on the RBP-ES interaction, expression profiles of RBP, PSI values of ES events, and protein-protein interactions to study the effects of RPB on ES events in differential gender groups.

**Fig. 5:**
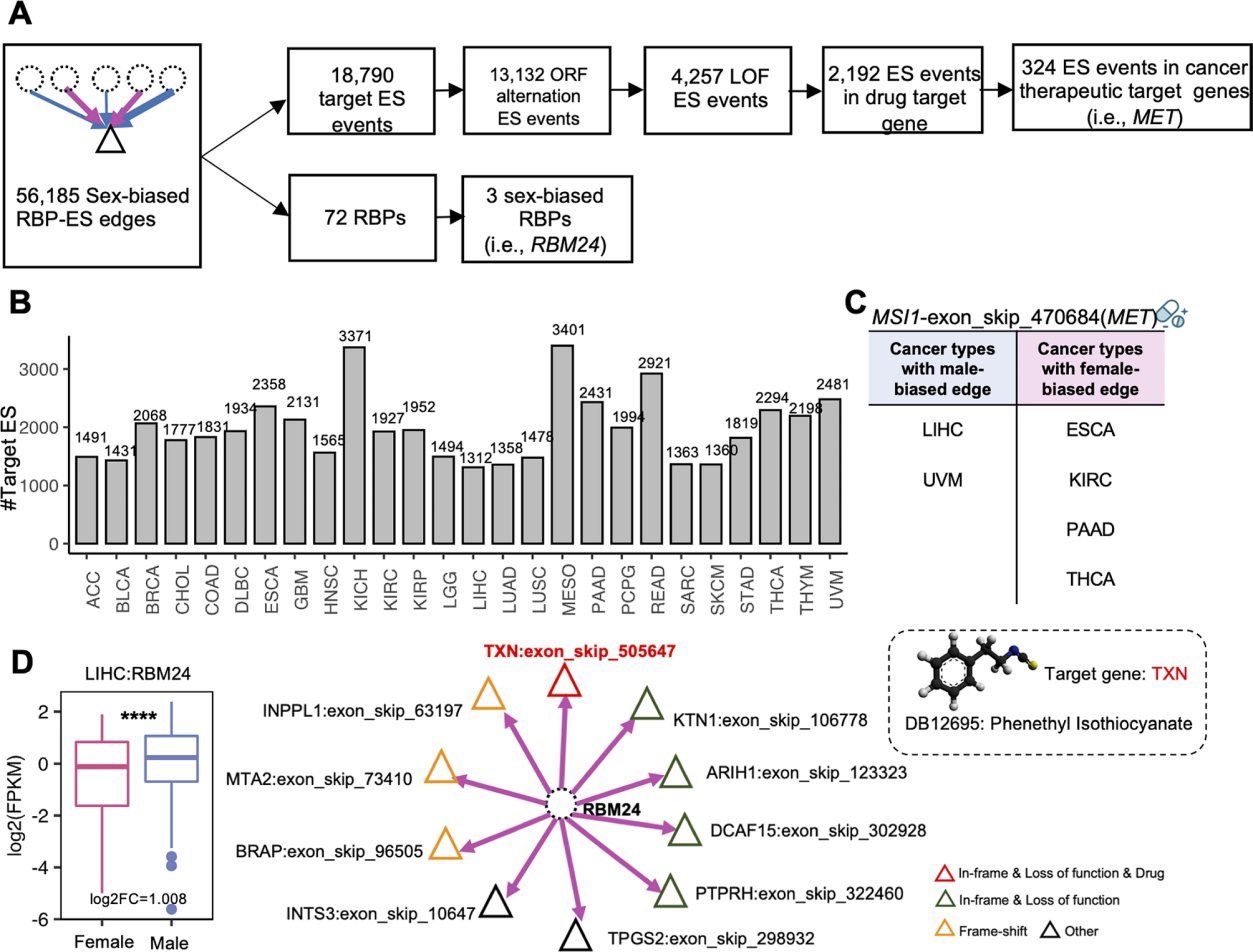
Sex-biased RBP-ES regulatory network analysis. **(A)** The pipeline to identify the functional ES events in the sex-biased RBP-ES network. **(B)** Number of RBP targeting ES events in sex-biased RBP-ES network of individual cancer type. **(C)** The sex-biased *MSI1*-exon_skip_470684 edges include two male-biased edges (LIHC, UVM) and four female-biased edges (ESCA, KIRC, PAAD, THCA). **(D)** Left: The expression of *RBM24* in LIHC. Right: *RBM24*-ES regulatory network in LIHC. (*p. adjusted < 0.05, **p.adjusted < 0.01, ***p.adjusted < 0.001, ****p.adjusted < 0.001)

Our analyses identified 56,185 sex-biased RBP-ES regulatory edges, including 18,790 ES events and 74 RBPs. Functional annotation of ES events showed that 13,132 targeting ES events could lead to open reading frame (ORF) alternation, of which 4,257 could lead to In-frame alternation of ORF and resulted in loss of protein function. Combined with drug information, we identified 2,192 targeting ES events appearing in drug target genes. 324 targeting ES events appeared in cancer therapeutic target genes (Fig. 5A and B). For example, *MET* lost the binding site of E3 ubiquitin ligase CBL through an exon 14 skipping event, resulting in an increased expression level of *MET*. *MET* amplification drives the proliferation of tumor cells [58, 59]. From the drug and disease annotation, Multiple tyrosine kinase inhibitors, such as crizotinib, cabozantinib, and capmatinib have been used to treat patients with *MET* exon 14 skipping. In addition, crizotinib, cabozantinib, and capmatinib have been approved by FDA to treat non-small cell lung cancer (NSCLC), non-hodgkin lymphoma (NHL), renal cell cancer (RCC), liver cancer. Our result found exon14 skipping (exon_skip_470684, chr7:116774880–116775111) of the *MET* gene showed the male-based regulation by *MSI1* in LIHC, UVM, and female-biased regulation by *MSI1* in ESCA, KIRC, PAAD, THCA. These results will help to design a personalized medical plan for the patient with *MET* exon 14 skipping (Fig. 5C).

For 72 RBPs related to sex-biased RBP-ES regulation, 3 RBPs were differentially expressed between male and female patients (Supplementary Table S6). For example, *RBM24* was sex-biased expressed in LIHC and targeted 10 ES events, 3 of which were frame-shift ES events and 5 were in-frame ES events. Among them, exon3 of *TXN* was the target of phenethyl isothiocyanate. We found *RBM24* also targeted *TXN* exon 3 skipping (exon_skip_505647, chr9:110244777–110244843) in CHOL, COAD, KIRC, KIRP, and PAAD female patients and PCPG and STAD male patients. The *TXN* gene encodes a protein involved in the process of the regulation of the cellular redox state [60]. In cancer cells, increased *TXN* expression increases proliferation and cell survival [61, 62]. Exon3 skipping of *TXN* can cause the in-frame alterations of *TXN-202*, leading to the loss function of “Beta strand”, “Chain”, “Disulfide bond”, “Domain”, “Helix”, “Modified residue”, “Mutagenesis”, and “Sequence conflict” to TXN protein(H9ZYJ2). Therefore, our results indicated that the downregulation of *RBM24* in female LIHC patients could cause the loss of normal function of *TXN* and inhibit cancer progression (Fig. 5D).

### TF-gene regulatory network reveals the sex-biased expression regulation between female and male patients

**Fig. 6:**
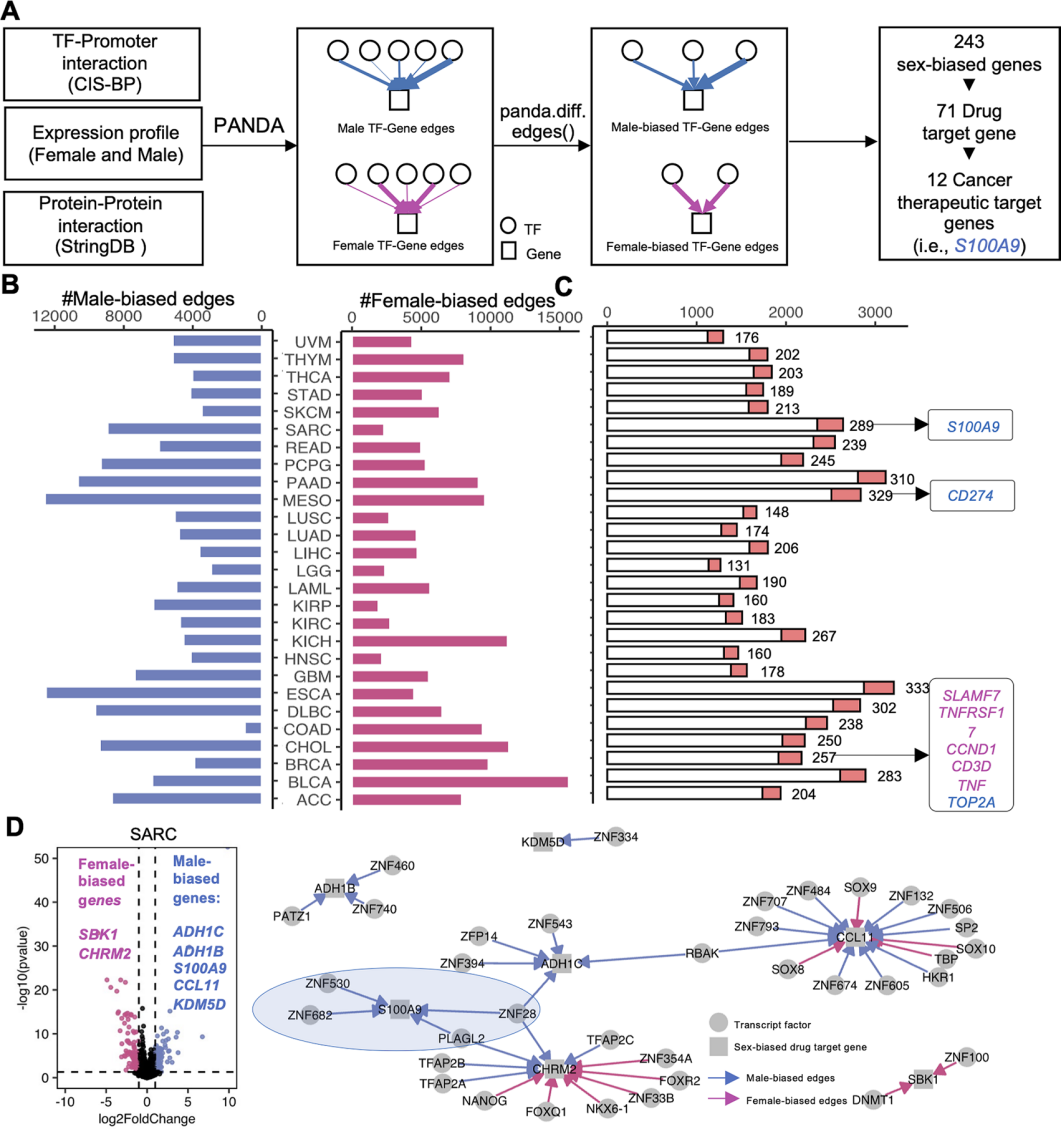
Sex-biased TF-Gene regulatory network analysis. **(A)** The pipeline to identify the cancer therapeutic target genes in the sex-biased TF-gene network. **(B)** The number of sex-biased edges for each cancer type. **(C)** The number of targeting genes (white bar) and drug-targeted genes (red bar) in the sex-biased TF-gene network for each cancer type. **(D)** Left: the volcano plot of sex-biased genes in SARC. Pink marked as female-biased genes (log2FC< − 1 and p.adjusted < 0.05), blue marked as male-biased genes (log2FC > 1 and p.adjusted < 0.05). Right: sex-biased TF-Gene regulatory network of 7 sex-biased drug targeted genes in SARC

Gene expression is controlled by complex networks of interacting factors. What’s more, the dysregulation of gene regulatory processes can lead to multiple diseases, including cancer. TF is one of the most important regulators of gene expression [63–65]. To explore the sex-biased TF-gene regulation, we constructed TF-gene regulatory networks based on TF-promoter interaction information, protein-protein interaction, and expression profiles for male and female patients, respectively (Fig. 6A). A total of 340,415 sex-biased edges were identified, including 169,132 male-biased edges and 171,283 female edges (Fig. 6B). 590 edges showed sex-biased regulation across more than 5 cancer types. Combined with differential analyses, we found that 243 targeting genes were differentially expressed between male and female patients. Besides, 71 sex-biased genes with sex-biased TF-gene regulations were also targeted by 491 drugs. 12 of the targeting sex-biased genes (*S100A9*,* SLAMF7*,* TNFRSF17*,* CCND1*,* CD274*,* TOP2A*,* HTR2B*,* CD3D*,* SSTR2*,* ORM2*,* ORM1*,* TNF*) have been reported previously as cancer therapeutic targets (Fig. 6C, Supplementary Fig. S8,Supplementary Table S7).

In SARC, a total of 289 targeting genes were reported as drug targets. Among them, *SBK1* and *CHRM2* were upregulated in male patients, and *ADH1C*,* ADH1B*,* S100A9*,* CCL11*,* and KDM5D* were upregulated in female groups. We found the female-biased genes owned more female-biased TF targeting edges and the male-biased genes owned more male-biased TF targeting edges. For example, *S100A9* was differentially expressed between male and female SARC patients (Fig. 2C). Our data suggested that four TFs targeting *S100A9* in the male group were responsible for the upregulation of *S100A9* in male patients and affected the cancer progression (Fig. 6D). This is consistent with the fact that TF targeting the gene promoter will promote the gene expression [66]. The sex-biased TF-gene network will help us to understand the sex difference regulation of the sex-biased genes in cancer.

### Sex-biased competitive endogenous RNA network

**Fig. 7:**
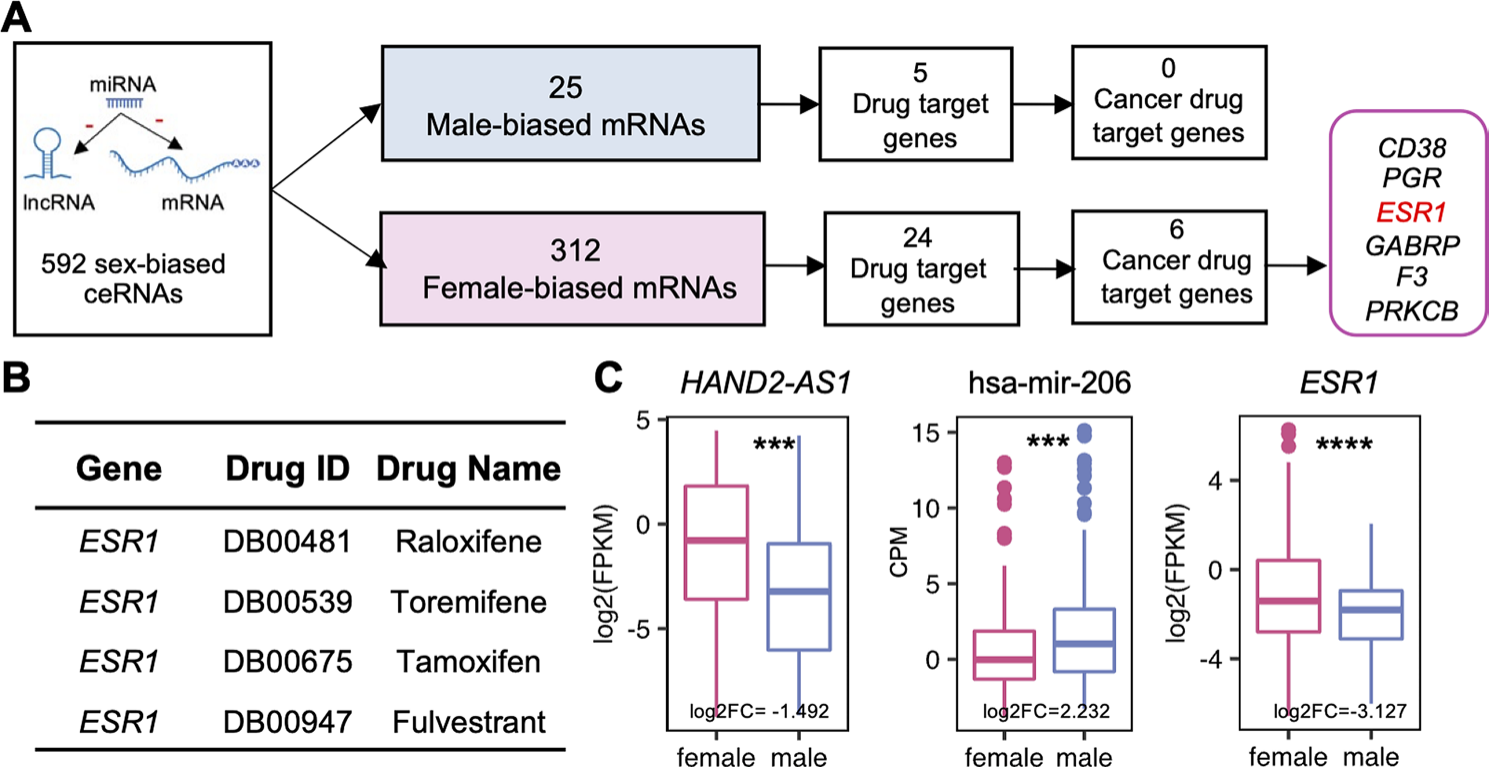
Sex-biased ceRNAs in human cancers. **(A)** The pipeline to identify the cancer therapeutic targets in sex-biased ceRNA network. **(B)**Related drug information of *ESR1*. **(C)** The expression of *HAND2-AS1*(lncRNA), hsa-mir-206(miRNA), and *ESR1*(mRNA) in SARC (*p. adjusted < 0.05, **p.adjusted < 0.01, ***p.adjusted < 0.001, ****p.adjusted < 0.001)

Competitive endogenous RNAs have revealed a new mechanism of interactions among diverse types of RNAs and play crucial roles in multiple biological processes and the development of neoplasms [67]. We constructed sex-biased ceRNAs regulatory networks for sex-biased lncRNA, miRNA, and mRNA. Our analyses found that 25 male-biased mRNAs and 312 female-biased mRNAs were involved in ceRNA regulation. Based on the drug information, 29 sex-biased mRNAs were drug-targeted genes and six (*CD38*,* PGR*,* ESR1*,* GABRP*,* F3*,* PRKCB*) of them were targeted by cancer therapeutic drugs (Fig. 7A,Supplementary Table S8). For example, *ESR1* is the target of raloxifene, toremifene, tamoxifen, and fulvestrant which are reported to prevent or treat breast cancer [33, 68–71] (Fig. 7B). *ESR1* mutations are a common cause of acquired resistance to the backbone of therapy in the metastatic hormone receptor-positive breast cancer [72–74]. A previous study reported that the microRNA hsa-mir-206 decreased endogenous ERα mRNA and protein levels in human MCF-7 breast cancer cells by acting through two specific hsa-mir-206 target sites within the 3′-UTR of the human ERα transcript [75]. In our study, the hsa-mir-206 was predicted to bind to both 3′-UTRs of *ESR1* and *HAND2-AS1*, indicating *HAND2-AS1* protects *ESR1* through competing for the miRNA binding sites of hsa-mir-206 in SARC female patients (Fig. 7C). In a word, our study identified potential sex-biased ceRNA regulation, providing insights for further research on the molecular mechanisms and potential prognosis biomarkers.

### Sex effects on cancer therapeutic target genes

**Fig. 8:**
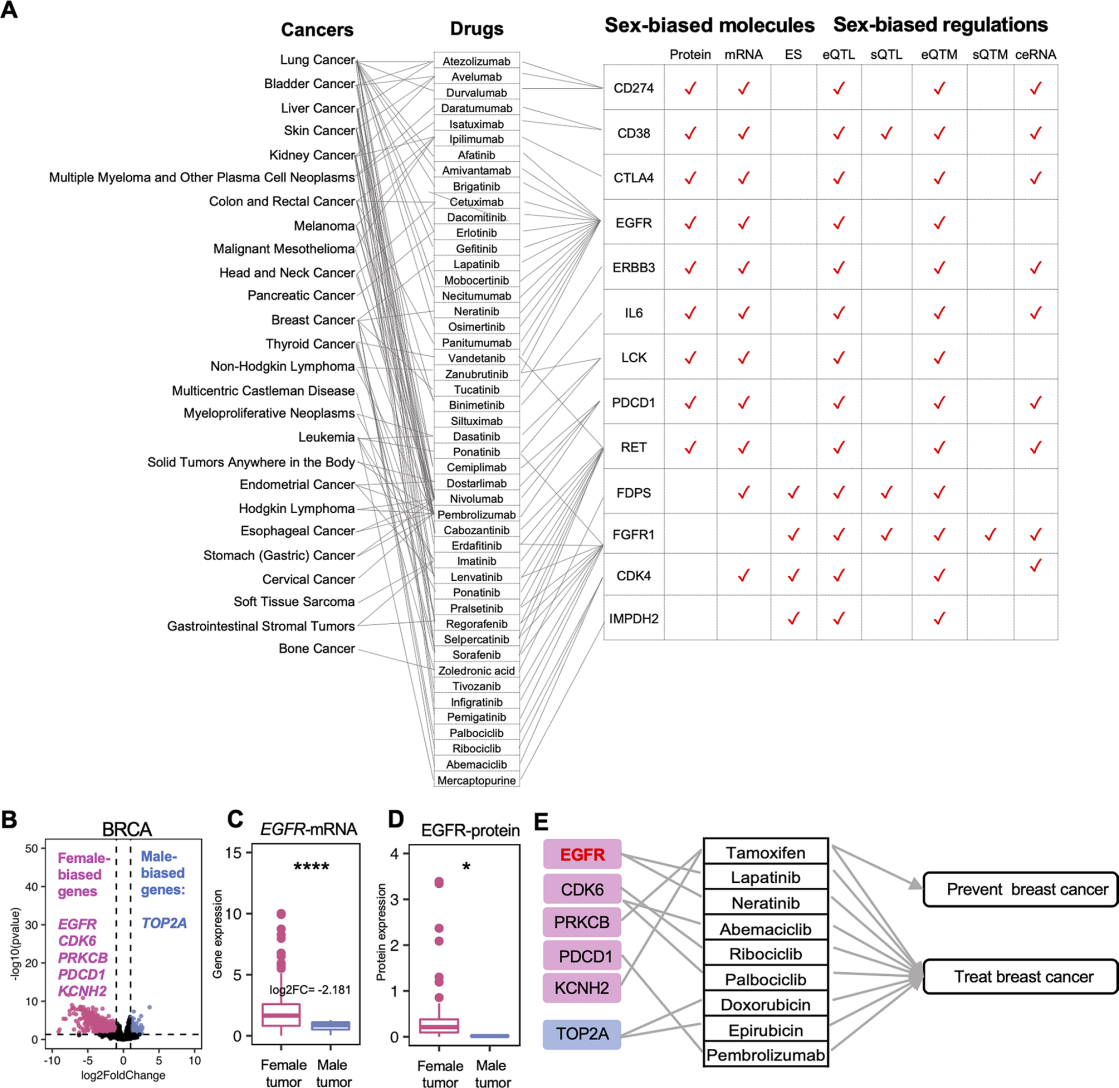
The sex-biased effect on cancer therapeutic targets. **(A)** Sex-biased cancer therapeutic drug target genes. The cancer-drug pairs were collected from the National Cancer Institute (https://www.cancer.gov/about-cancer/treatment/drugs/cancer-type) and the drug-gene pairs were collected from DrugBank. **(B)**The volcano plot of sex-biased genes in BRCA. Pink marked as female-biased genes (log2FC<–1 and p.adjusted < 0.05), blue marked as male-biased genes (log2FC > 1 and p.adjusted < 0.05). **(C)** The gene expression of *EGFR* in BRCA. **(D)** The protein expression of EGFR in BRCA. **(E)** Cancer-drug-sex-biased gene information in BRCA (*p. adjusted < 0.05, **p.adjusted < 0.01, ***p.adjusted < 0.001, ****p.adjusted < 0.001)

To investigate the clinical implications of sex-biased molecular signatures, we focused on 2,526 therapeutic targets in DrugBank, including 205 cancer therapeutic targets of FDA-approved drugs. Across the various molecule dimensions we examined, we found that 131 drug target genes were associated with at least one type of sex-biased signature, and 126 were cancer therapeutic target genes (Supplementary Table S9). Among these genes, nine genes (*CD274*,* CD38*,* CTLA4*,* EGFR*,* ERBB3*,* IL6*,* LCK*,* PDCD1*,* and RET*) showed sex differences not only in mRNA expression but also in protein levels. These genes also showed sex-biased cis-eQTLs and cis-eQTMs regulation, indicating that sex differences at the transcriptome level are partially the result of corresponding sex-biased epigenetic and genetic regulations. Besides, four sex-biased exon skipping events in cancer therapeutic target genes (*FDPS*,* FGFR1*,* CDK4*,* and IMPDH2*) could cause the loss of partial protein function, which may contribute to a differential drug response rate in male and female patients (Fig. 8A). Moreover, we found six sex-biased genes (*EGFR*,* CDK6*,* PRKCB*,* PDCD1*,* KCNH2*,* FCGR3B*,* and TOP2A)* in BRCA were the therapeutic targets of breast cancer (Supplementary Fig. S9). *EGFR* is one of the first identified important targets of these novel antitumor agents [76]. Two drugs (Lapatinib and neratinib) for the treatment of breast cancer have been evaluated in several studies. Our result showed *EGFR* had differential expression both at the transcriptome level and proteome level, which are particularly important in determining sex-biased drug response rates (Fig. 8B-E). These results highlight the clinical importance of sex-biased molecular signatures.

Furthermore, from a manual review of the abstracts, we found that there are 31 documentaries supporting 40 genes showing different patterns between females and males. Among them, 13 genes (AFP, ASNS, BEX4, BRAF, ESR1, FST, KIT, NR3C1, STAT3, TP53, TRPA1, AR, FST) were reported to show sex differences in cancer (**Supplementary Table 10**). Among them, ESR1 is involved in a sex-biased competitive endogenous RNA network, ESR1 competing for the miRNA binding sites of hsa-mir-206 in SARC female patients, TP53 shows higher mutation frequency in male LIHC patients, which is consistent with the previous research (Fig. 7, Supplementary Fig. S5A). These findings underscore the importance of considering sex-specific differences in cancer research, as they shed light on distinct genetic patterns and potential molecular mechanisms that may contribute to gender-specific variations in cancer development and progression.

## Discussion

Clinical cancer research indicates that men and women showed different responses to drug treatment [77, 78]. Understanding the sex-biased regulation related to cancer therapeutic target genes will help the basic experimental researchers or physicians develop personalized treatment plans. In this study, we performed comprehensive analyses to explore sex-associated molecular differences in cancer patients. 4,328 genes were found to have at least one kind of sex-biased signature and functionally enriched in oncogenic, immune response, cell proliferation, and cancer-related pathways. These enrichments imply that the sex-biased signatures participate in the cancer progression, which helps to systematically explain the sex differences in cancer and facilitates antitumor studies and clinical practices. Analyzing these sex-biased regulations of cancer types will help to achieve a molecular-level understanding of how sex signatures affect the behavior of different cancer types.

Previous studies focus on the mutation and gene expression between the male and female groups [8, 10–12](Supplementary Table S11). However, recently the small molecule splicing modulators are currently in clinical trials [79, 80]. This paper investigates sex-biased exon skipping events and sex-biased RNA-binding protein-exon skipping (RBP-ES) regulatory network analyses. Differential analysis revealed 121 sex-biased exon skipping events, with 70 causing alterations in the open reading frame. Additionally, RBP-ES network analysis identified 324 RBP-targeted exon skipping events in cancer therapeutic target genes associated with sex-biased RBP-ES regulation. Although these events did not exhibit PSI values between male and female patients, we identified three sex-biased RBPs in the network related to functional exon skipping events, resulting in ORF alterations and partial protein loss of function. We hypothesize that these sex biased RBPs may impact drug resistance and cancer progression by regulating exon skipping events. Furthermore, sQTM and sQTL analyses were conducted, revealing differential regulation patterns of exon skipping between males and females. Our database is the first one incorporating alternative splicing related to sex-biased regulation, offering potential sex-related alternative splicing markers.

Currently, male and female patients with the same cancer type are often treated similarly regardless of gender. We must accept that these sex differences in cancer biology will affect individual responses to therapy. Our study reported 126 cancer therapeutic target genes with sex-biased molecules and comprehensively compared the regulatory networks between male and female patients. Understanding the role of sex differences is critical not only for understanding health and disease but also for personalized and precision medicine [81, 82]. The sex-related genes found in each category can provide tailored therapy and better suggestions for drug selection. All the above information is included on the SexAnnoDB website and is available for download. We believe that SexAnnoDB will be routinely used in cancer studies to better understand cancer pathogenesis, progression, and biology.

### Perspectives and significance

In summary, Understanding the sex disparities in gene expression and mutation frequencies could have significant implications for personalized medicine and the development of targeted therapies tailored to specific patient populations. Our work identified 4,328 genes exhibiting sex-biased signatures, with 126 of these genes being implicated as cancer therapeutic targets. SexAnnoDB provided a valuable resource and reference for extensive annotations of sex-difference-related regulations of various molecules in cancer.

### Electronic supplementary material

Below is the link to the electronic supplementary material.

Supplementary Material 1

Supplementary Material 2

Supplementary Material 3

## Data Availability

All annotation results are available from the SexAnnoDB website (https://ccsm.uth.edu/SexAnnoDB). The code is available on GitHub (https://github.com/MengyuanYang1/SexAnnoDB).
