# Supplementary material for: SexAnnoDB, a knowledgebase of sex-specific regulations from multi-omics data of human cancers
Source: Biol Sex Differ. 2024 Aug 22;15:64. doi: 10.1186/s13293-024-00638-8 (PMC11342657; doi:10.1186/s13293-024-00638-8)
Supplement: Supplementary file 2 — Supplementary Material 2 [file 13293_2024_638_MOESM2_ESM.docx]

**SexAnnoDB, a knowledgebase of sex-specific regulations from multi-omics data of human cancers**

Mengyuan Yang^1, *^, Yuzhou Feng^,2,3^, Jiajia Liu^4^, Hong Wang^1^, Sijia Wu^5^, Weiling Zhao^4^, Pora Kim^4, *^, Xiaobo Zhou^4, *^

*^1^* *School of Life Sciences, Zhengzhou University, Zhengzhou, 450001, China*

*^2^ West China Biomedical Big Data Center, West China Hospital, Sichuan University, Chengdu 610041, China*

*^3^ Med-X Center for Informatics, Sichuan University, Chengdu 610041, China*

*^4^ Center for Computational Systems Medicine, McWilliams School of Biomedical Informatics, The University of Texas Health Science Center at Houston, Houston, 77030, USA*

*^5^ School of Life Sciences and Technology, Xidian University, Xi’an, 710126, China*

*Corresponding author(s).

E-mail:[Xiaobo.Zhou@uth.tmc.edu](mailto:Xiaobo.Zhou@uth.tmc.edu)(Zhou X), [Pora.Kim@uth.tmc.edu](mailto:Pora.Kim@uth.tmc.edu)(Kim P),[mengyuanyang@zzu.edu.cn](mailto:mengyuanyang@zzu.edu.cn) (Yang M)

**Supplementary methods**

This supplement primarily provides elaboration the performed of PANDA in RNA binding protein-exon skip events regulatory network using 8 RBPs knockdown/out RNA-seq data and matched eCLIP data.

**Data Collection**

Initially, we obtained HepG2 shRNA-seq, control HepG2 RNA-seq, and matched eCLIP data for 8 RBPs (*HNRNPL, HNRNPA1, HNRNPC, SRSF1, PCBP1, U2AF2, FUS, PTBP1*) from ENCORE (<https://www.encodeproject.org/encore-matrix/?type=Experiment&status=released&internal_tags=ENCORE>). To identify regulations related to RNA-binding protein (RBP)-mediated exon skipping, we initially preprocessed shRNA-seq and control RNA-seq data using trimmomatic 0.39 for quality trimming, STAR 2.7.10b for alignment, and samtools 1.15 along with Spladder 3.0.4 for post-alignment processing to quantify gene expression and detect alternative splicing events. Differential exon skipping events were identified using the Wilcoxon test, followed by Benjamini-Hochberg correction to control the false discovery rate. Exon skipping events were considered significant if the mean differential percent spliced-in value exceeded 0.1, with a p.adjust threshold of 5%. Finally, bedtools v2.31.1 was utilized to determine exon-skipping events targeted by each RBP within the skipped exon regions using the RBP target narrow peak region obtained from ENCORE(**Supplementary Fgure 2**).

*trimmomatic PE -threads 8 R1.fastq.gz R2.fastq.gz R1_ter.fastq R1_ter_unpaired.fastq R2_ter.fastq R1_ter_unpaired.fastq ILLUMINACLIP:TruSeq3-SE.fa:2:30:10 LEADING:3 TRAILING:3 SLIDINGWINDOW:4:20 MINLEN:50*

*STAR --runThreadN 8 --genomeDir STAR_index_v39 --readFilesCommand cat --readFilesIn R1_ter.fastq R2_ter.fastq --outFileNamePrefix STAR_out/sample1 --outSAMtype BAM SortedByCoordinate --outBAMsortingThreadN 16 --quantMode TranscriptomeSAM GeneCounts --outSAMstrandField intronMotif --outSAMattributes NH HI NM MD AS XS*

*samtools index sample1.bam*

*spladder build -o ./spladder_out/ -a gencode.v39.annotation.gtf -b sample1.bam,sample2.bam,sample3.bam,sample4.bam*

**Performed RBP-ES regulation network using PANDA**

We performed PANDA analysis on RNA-seq data from knockdown/out experiments involving 8 RBPs to construct regulatory networks. This analysis utilized RBP-ES interactions from eCLIP data, PSI values from RBP knockdown and control groups, and protein-protein interaction data from StringDB v11.5. We selected the top 10% predicted edges by PANDA and assessed the quality of the predicted networks using AUC-ROC statistics (AUC). The code is available at <https://github.com/MengyuanYang1/SexAnnoDB>.
