# Supplementary material for: SexAnnoDB, a knowledgebase of sex-specific regulations from multi-omics data of human cancers
Source: Biol Sex Differ. 2024 Aug 22;15:64. doi: 10.1186/s13293-024-00638-8 (PMC11342657; doi:10.1186/s13293-024-00638-8)
Supplement: Supplementary file 3 — Supplementary Material 3 [file 13293_2024_638_MOESM3_ESM.docx]

**SexAnnoDB, a knowledgebase of sex-specific regulations from multi-omics data of human cancers**

Mengyuan Yang^1, *^, Yuzhou Feng^,2,3^, Jiajia Liu^4^, Hong Wang^1^, Sijia Wu^5^, Weiling Zhao^4^, Pora Kim^4, *^, Xiaobo Zhou^4, *^

*^1^* *School of Life Sciences, Zhengzhou University, Zhengzhou, 450001, China*

*^2^ West China Biomedical Big Data Center, West China Hospital, Sichuan University, Chengdu 610041, China*

*^3^ Med-X Center for Informatics, Sichuan University, Chengdu 610041, China*

*^4^ Center for Computational Systems Medicine, McWilliams School of Biomedical Informatics, The University of Texas Health Science Center at Houston, Houston, 77030, USA*

*^5^ School of Life Sciences and Technology, Xidian University, Xi’an, 710126, China*

*Corresponding author(s).

E-mail:[Xiaobo.Zhou@uth.tmc.edu](mailto:Xiaobo.Zhou@uth.tmc.edu)(Zhou X), [Pora.Kim@uth.tmc.edu](mailto:Pora.Kim@uth.tmc.edu)(Kim P),[mengyuanyang@zzu.edu.cn](mailto:mengyuanyang@zzu.edu.cn) (Yang M)

**Supplementary figures**


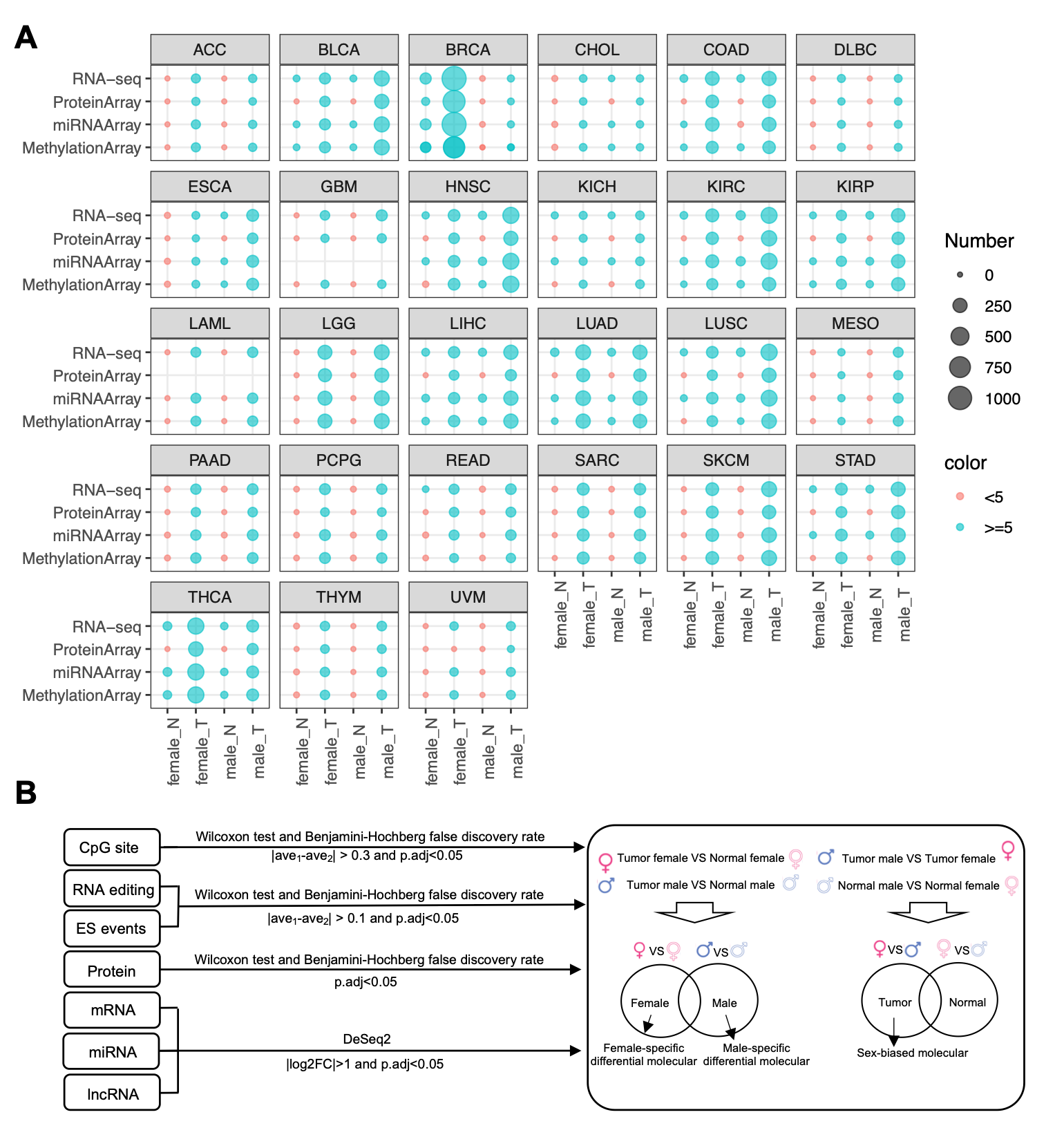


**Supplementary figure 1.** Sex-biased signatures and Sex-specific differental signatures. **(A)** The sample size of each group used to identify Sex-biased signatures. **(B)** The pipeline to identify the sex-biased signatures and sex-specific differential signatures.

**
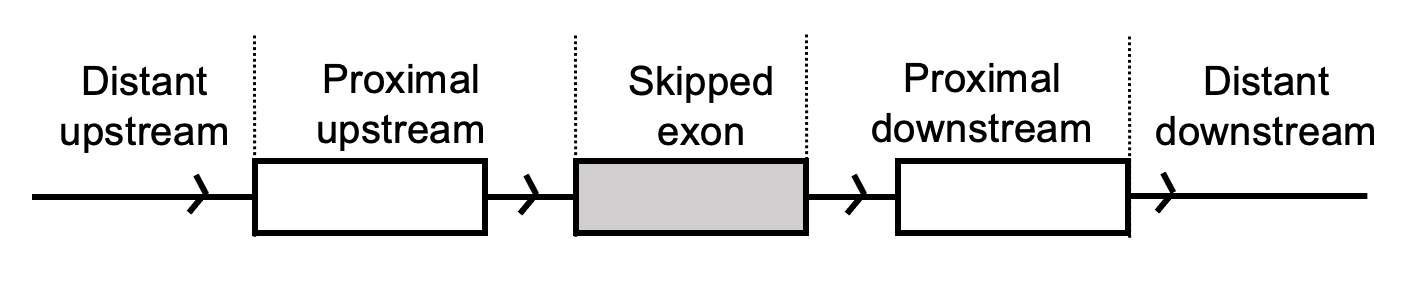
**

**Supplementary figure 2. The structure of exon skipping events**


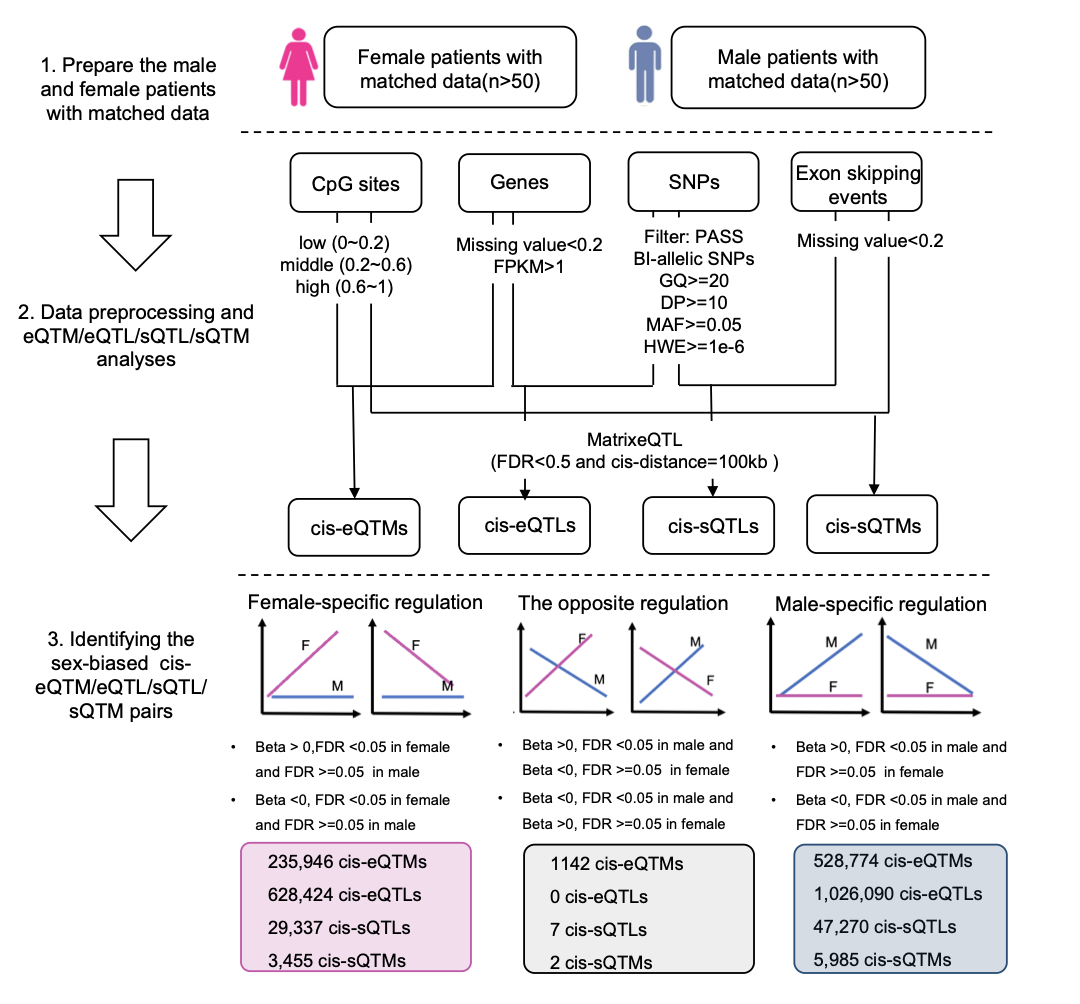


**Supplementary figure 3. The pipeline to identify sex-biased cis-eQTM eQTL/sQTL/sQTM pairs**


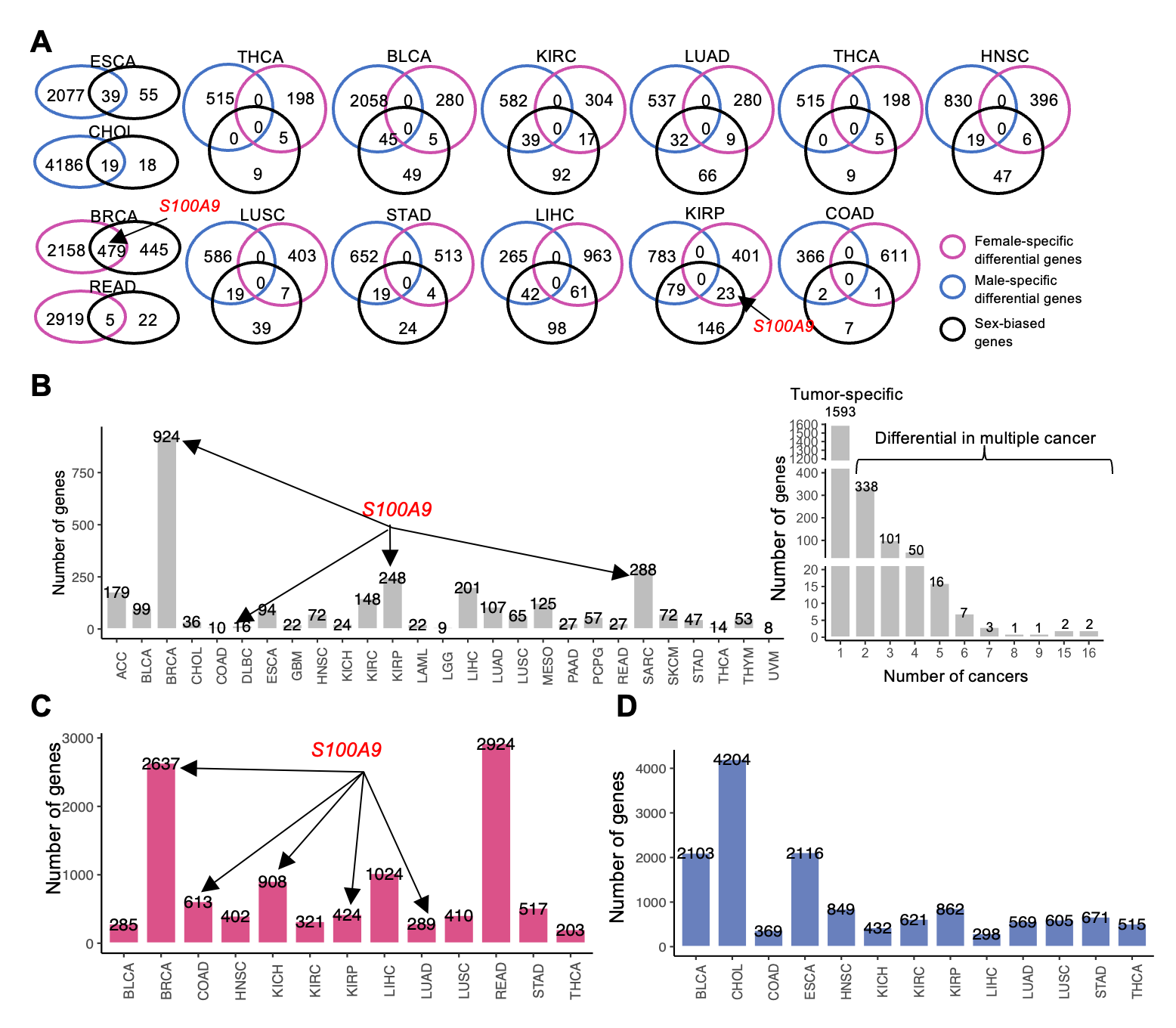


**Supplementary figure 4 Sex-biased mRNAs and sex-specific differential mRNAs.** (**A**) Depicts the overlap of sex-biased genes, female-specific differential genes, and male-specific differential genes across 27 cancer types. (**B**) On the left, it shows the distribution of sex-biased genes in 27 cancer types. On the right, it displays the overall distribution of sex-biased genes across multiple cancers. (**C**) Represents the number of differential genes between female tumors and female normal tissues. (**D**) Represents the number of differential genes between male tumors and male normal tissues.


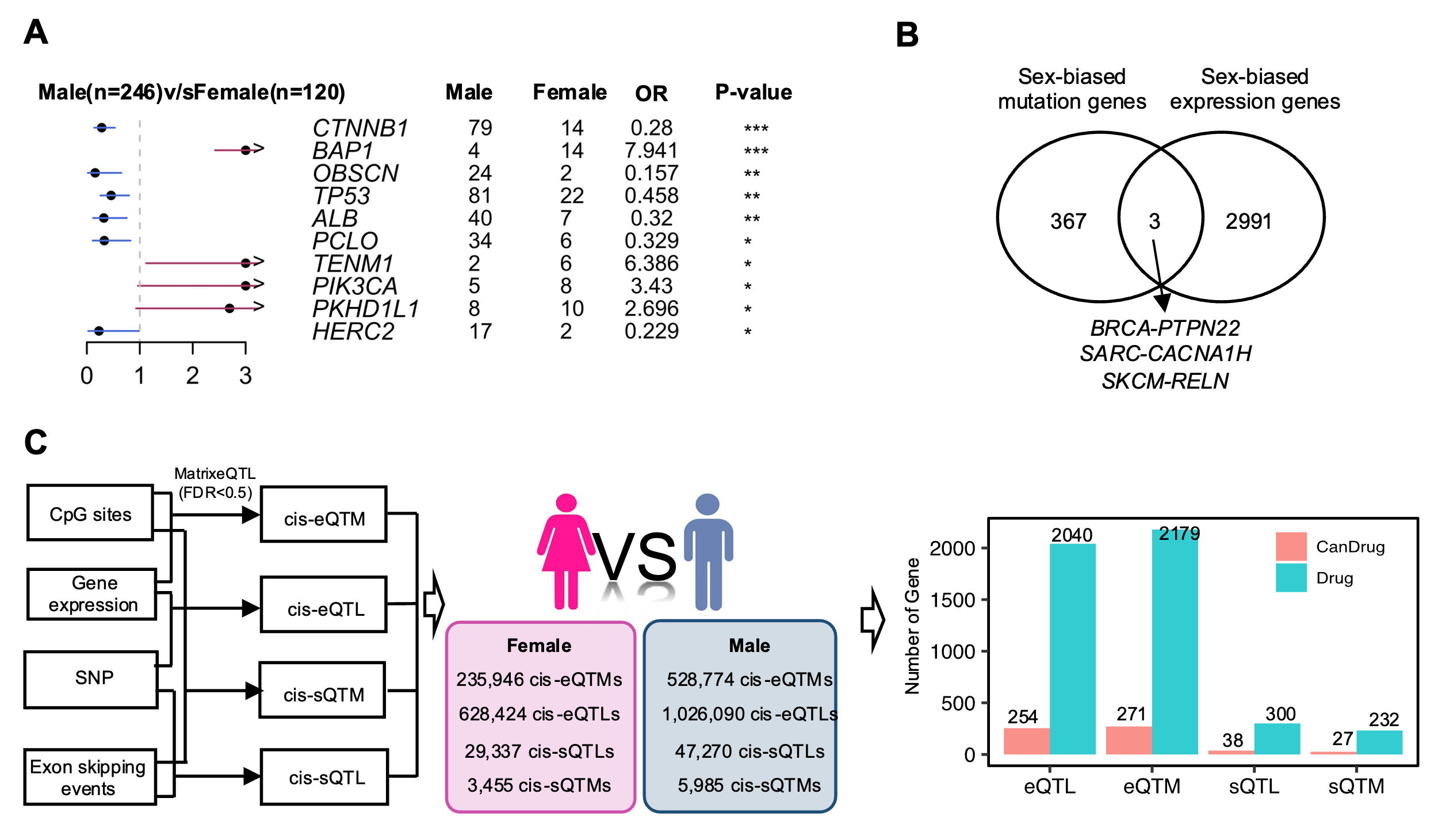


**Supplementary figure 5. Sex-biased mutation.** **(A)** The sex-biased mutation genes in LIHC. Odd ratio(OR) with 95% CI, OR = 1 represent no effects, OR<1 represent male-biased mutation, OR>1 represent female-biased mutation. **(B)** The overlap between sex-biased mutation genes and sex-bised expression genes in all cancer.**(C)** The pipeline of eQTL/sQTL/eQTM/sQTM analyses.

**
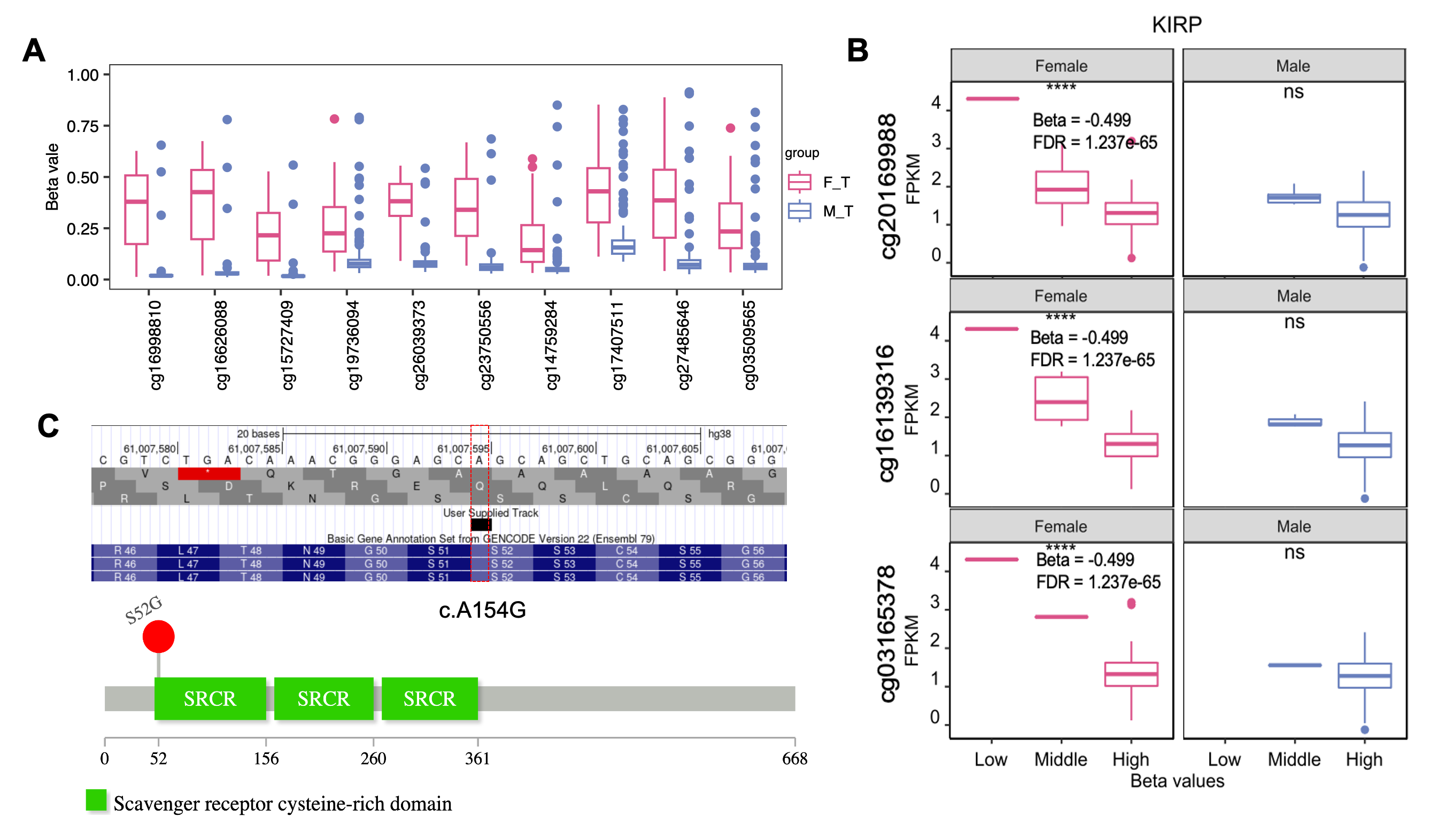
**

**Supplementary figure 6. Sex-biased menthylation and RNA editing. (A)** the boxplot of 10CpGsites in SRPX. **(B)**The sex-biased eQTM pairs related to *S100A9****.*(C)** RNA editing site in SRPX. (*FDR <0.05, ** FDR <0.01, *** FDR <0.001, **** FDR <0.0001).

**Supplementary figure 7. An evaluation of PANDA’s performance in RBP-ES regulation**.

**
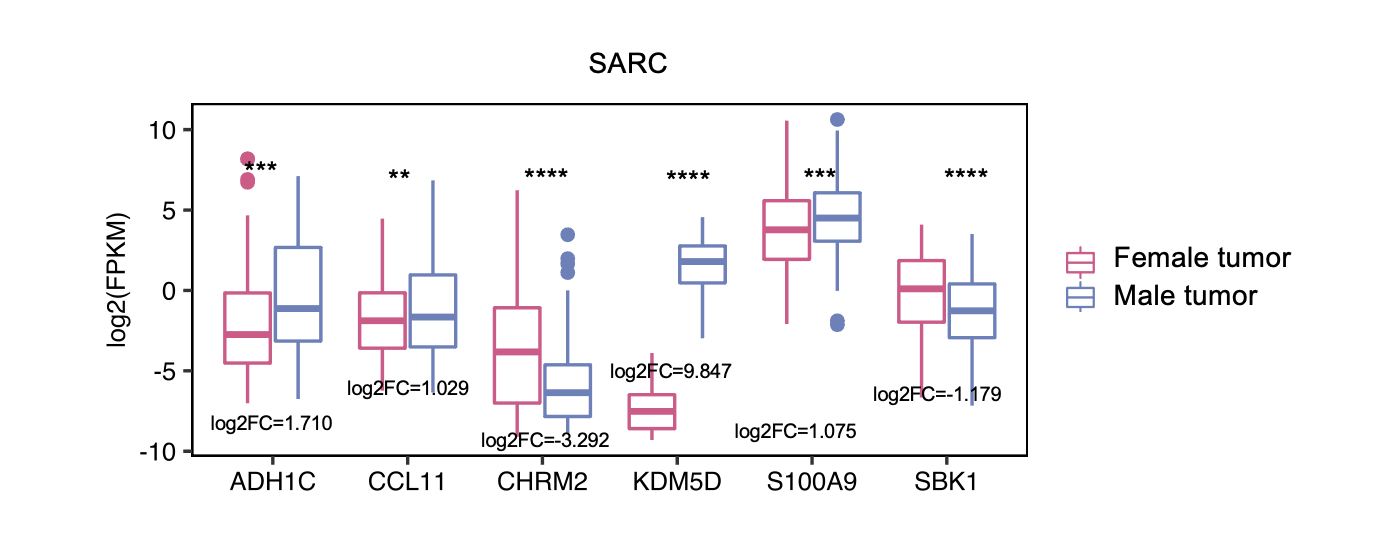
**

**Supplementary figure 8. The sex-biased genes of TF-gene regulatory network in SARC**

(*p. adjusted <0.05, **p.adjusted <0.01, ***p.adjusted <0.001, ****p.adjusted <0.0001).


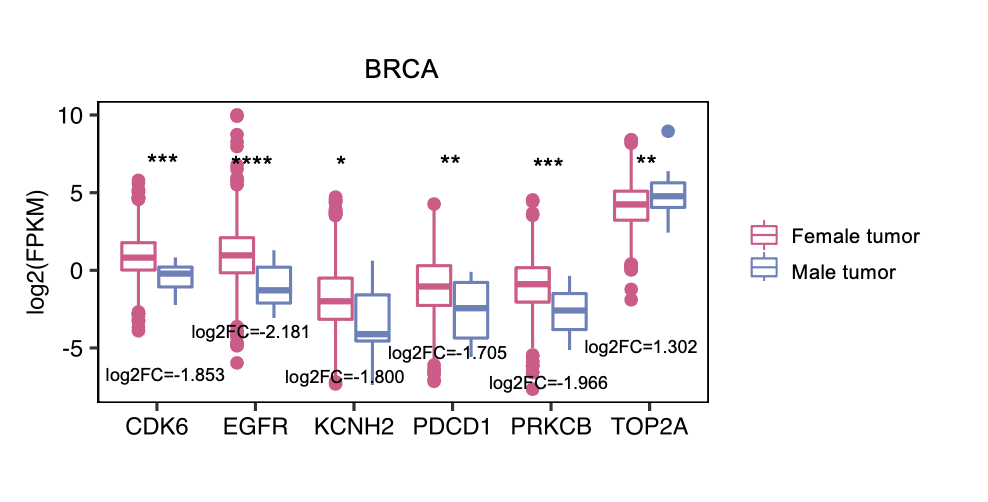


**Supplementary figure 9. The sex-biased cancer therapeutic target genes in BRCA**

(*p. adjusted <0.05, **p.adjusted <0.01, ***p.adjusted <0.001, ****p.adjusted <0.0001).
